# MBQG-Net: A Multi-Scale Bidirectional Query-Guided Gated Network for Aero-Engine Remaining Useful Life Prediction

**DOI:** 10.3390/e28101082

**Published:** 2026-09-30

**Authors:** Xiao Hu, Hao Qi, Jing Yu, Chengwu Lu, Lingli Zhang

**Affiliations:** 1 School of Mathematics and Statistics, Chongqing Jiaotong University, Chongqing 401331, China; 622240150012@mails.cqjtu.edu.cn; 2 Chongqing Key Laboratory of Statistics Optimization and Complex Data, Chongqing University of Arts and Sciences, Chongqing 402160, China; jyu2720@163.com; 3 Department of Mathematics and Statistics, The University of North Carolina at Charlotte, Charlotte, NC 28223, USA; hqi1@charlotte.edu; 4 School of Mathematics and Big Data, Chongqing University of Education, Chongqing 400065, China

**Keywords:** aircraft engine, remaining useful life prediction, multi-scale bidirectional temporal convolution, query–value temporal attention, stacked bidirectional GRU, asymmetric regression

## Abstract

Remaining Useful Life (RUL) prediction for aircraft engines is a critical task in prognostics and health management, aiming to extract degradation information from multi-sensor operational data to support maintenance scheduling and operational risk management. Existing hybrid temporal models have explored various combinations of convolutional networks, temporal convolutions, recurrent networks, and attention mechanisms; however, there remains room for further coordination regarding the positional roles of different modules within fixed windows, bidirectional context organization, and training objectives. To address this, we propose a Multi-scale Bidirectional Query-Guided Gated Network, MBQG-Net. MBQG-Net employs one-dimensional convolution and parallel bidirectional dilated convolutions to extract local and multi-range temporal features, and utilizes multi-head Query–Value temporal attention to generate window-level context-enhanced representations prior to recurrent state aggregation. The sequence enhanced by Query-based temporal weighting and Value aggregation is then fed into a stacked bidirectional GRU for subsequent bidirectional gated state aggregation. During training, an asymmetric weighted mean squared error is adopted, assigning higher weights to RUL overestimation errors. MBQG-Net was evaluated on all four official test sets (FD001–FD004) of the NASA C-MAPSS dataset under a unified experimental protocol. It achieved the strongest overall regression performance on FD001 and FD003 and maintained competitive overall conventional regression performance on the multi-condition FD002 and FD004 subsets. The model obtained the lowest NASA Score among the compared methods on all four subsets, although the difference on FD002 was marginal. On FD004, MBQG-Net achieved a NASA Score of 930.9088, representing a 28.27% reduction relative to the second-best result. Experimental results demonstrate that MBQG-Net maintains a favorable balance between conventional regression accuracy and direction-sensitive error control across different operating-condition and fault-mode settings, offering a structurally clear hybrid temporal modeling solution for multi-sensor aircraft engine RUL prediction.

## 1. Introduction

The aeroengine is the core component of the power system of an aircraft. Its internal components operate under complex conditions such as high temperature, high pressure, and strong vibration for an extended period, and their performance and status gradually deteriorate during the service process. With the development of sensor technology, industrial Internet of Things, and intelligent operation and maintenance technology, during the engine operation, multiple-source state monitoring data such as temperature, pressure, rotational speed, and flow rate can be continuously accumulated, providing a data foundation for equipment health status assessment and intelligent maintenance.

Prognostics and Health Management (PHM) utilizes condition monitoring, fault prediction, and health assessment to provide technical support for the operation management and maintenance decisions of complex equipment [1]. Among them, the remaining useful life (RUL) represents the time remaining for the equipment to continue operating from its current state until it fails functionally, and is an important quantitative basis connecting health status assessment with predictive maintenance decisions [2,3].

In terms of maintenance strategies, traditional time-based maintenance (Time-Based Maintenance, TBM) mainly schedules maintenance based on preset operating cycles, while condition-based maintenance (Condition-Based Maintenance, CBM) formulates maintenance plans based on equipment monitoring status and performance changes [4]. On this basis, predictive maintenance (Predictive Maintenance, PdM) further combines state monitoring information and future state predictions of the equipment to plan maintenance activities in advance [5]. Accurate RUL prediction can provide references for maintenance timing arrangement, spare parts configuration, and operation risk management, helping to reduce sudden failures, unplanned downtime, and safety risks and economic losses caused by premature maintenance.

The existing RUL prediction methods can generally be classified into model-driven methods, data-driven methods, and hybrid methods that combine the two. Model-driven methods establish mathematical models based on the equipment structure, damage mechanism, and degradation laws, and have relatively clear physical meanings. However, there is complex coupling among the internal components of aircraft engines, and their operating status is also affected by environmental changes, measurement noise, and degradation uncertainties. Therefore, it is often difficult to accurately establish a failure mechanism model and transfer it to different equipment and operating conditions. Data-driven methods do not need to fully describe the internal degradation mechanism of the equipment, but instead use historical monitoring data to learn the mapping relationship between the equipment status and health level or RUL. This has become an important technical route for industrial predictive maintenance. Early data-driven research mainly relied on statistical degradation models, health index construction, signal decomposition, trajectory matching, and shallow machine learning. These methods can introduce domain knowledge and degradation priors into the prediction process, but their performance is usually affected by the quality of artificial features, decomposition strategies, and parameter settings, and the feature generation process is also difficult to be unified and optimized end-to-end with the final RUL regression target.

As the monitoring dimensions and data scale of aircraft engines increase, RUL prediction gradually becomes a high-dimensional, multi-variable, nonlinear and strongly time-dependent sequential regression problem. Different sensors record the state changes of different engine components and operating processes, and their response patterns, variation amplitudes and degradation sensitivities vary significantly. The engine degradation process includes both local fluctuations within adjacent operating cycles and gradual cumulative state changes over a longer time range, making it difficult for models relying solely on manual features or a single time scale to fully describe the complex degradation patterns. Deep neural networks can directly learn hierarchical feature representations from multi-sensor sequences, and thus have gradually become an important research direction in data-driven RUL prediction [6]. The convolutional structure is suitable for extracting local time patterns; the temporal convolutional network (Temporal Convolutional Network, TCN) expands the sequence receptive range through dilated causal convolution and residual structures [7]. Long short-term memory networks (Long Short-Term Memory, LSTM) and gated recurrent units (Gated Recurrent Unit, GRU) aggregate historical sequence information through gated state updates [8,9]. The attention mechanism can implement non-uniform weighting for different time positions or feature representations based on the input content [10].

Based on the above infrastructure, existing studies have further explored various combinations of convolutional networks, time convolution, recurrent networks, and attention mechanisms, and have constructed different RUL prediction frameworks from perspectives such as local feature extraction, time context modeling, multi-sensor fusion, and sequence state aggregation. However, from the modeling requirements of fixed-length multi-sensor windows, local changes, cross-interval degradation information, and the relative contributions at different time positions still need to be coordinated in a unified information flow. Firstly, in the fixed window, both short-term fluctuations and long-range degradation trends are present, and it is necessary to integrate the forward and backward contexts of different time ranges in the compressed sequence. Secondly, when the time features extracted by the convolution module are passed to the recurrent network, the contributions of different time positions to the current RUL prediction are not consistent, and a window-level context-enhanced representation needs to be formed before the recurrent state aggregation. For this intermediate enhancement stage, the primary requirement is to assign adaptive importance to temporal positions within the current degradation window and aggregate their corresponding feature representations, rather than to explicitly model pairwise similarities between all temporal positions. Finally, underestimation of RUL usually leads to maintenance acceleration, while overestimation of RUL may cause maintenance delay. The two types of prediction errors have different engineering consequences, and thus the training objective also needs to reflect the difference in error direction.

To address the aforementioned issues, this paper proposes a multi-scale bidirectional query-guided gated network (Multi-scale Bidirectional Query-Guided Gated Network, MBQG-Net) for predicting the remaining useful life of aircraft engines. The model first uses one-dimensional convolution to perform local temporal encoding and sequence compression on the multi-sensor sliding window. Subsequently, the multi-scale bidirectional temporal convolution module (MS-BiTCN) integrates forward and backward temporal responses through parallel dilated-convolution branches with different dilation rates. Between convolutional representation learning and recurrent state aggregation, multi-head Query–Value temporal attention uses Query to generate temporal-position weights and aggregates the corresponding Value representations to form a window-level context. After output mapping, the context is injected into the original temporal sequence through a residual connection, and the enhanced sequence is further processed by the stacked BiGRU for bidirectional gated state aggregation. During training, an asymmetric weighted mean squared error is adopted to impose a stronger penalty on RUL overestimation errors and thereby reflect the different directions of prediction error. To assess the model under different operating-condition and fault-mode complexities, experiments are conducted on all four C-MAPSS sub-datasets, covering both single- and multi-condition settings as well as one- and two-fault-mode scenarios.

The main contributions of this paper are summarized as follows:

1. Propose a hybrid time-series modeling framework MBQG-Net for the RUL prediction of aero-engines. This framework jointly learns convolutional temporal features, window-level context representations, and bidirectional recurrent states from multi-sensor sliding windows, achieving end-to-end RUL regression.
2. Construct a joint representation structure combining MS-BiTCN and multi-head QV temporal attention. MS-BiTCN employs parallel bidirectional dilated-convolution branches with different dilation rates to extract temporal responses at multiple scales; the QV temporal attention forms a window-level context through Query-based temporal scoring and Value aggregation, without introducing an independent Key projection or pairwise Query–Key similarity calculation, and injects the mapped context into the temporal sequence through a residual connection before the stacked BiGRU. This organization allows the subsequent bidirectional gated state aggregation to operate on a context-enhanced temporal representation.
3. Design an asymmetric weighted MSE training objective that is sensitive to the overestimation error of RUL. This objective implements differentiated weighting based on the direction of prediction errors, imposing stronger constraints on overestimation errors of RUL, so that the training objective aligns with the asymmetric penalty direction of the NASA Score.

## 2. Related Work

Regarding the effective utilization of multi-sensor degradation information, the existing research on RUL prediction for aircraft engines mainly focuses on explicit degradation characterization and feature construction, deep hybrid time series modeling, and attention-enhanced context modeling. Different methods have formed different information processing flows in terms of input representation, time context extraction, attention target objects, and aggregation methods of recurrent states. This section organizes the research most relevant to this paper along the above technical route and further explains the structural positioning of MBQG-Net within the existing method framework.

### 2.1. Degradation Characterization and Feature Construction Methods

A class of RUL prediction methods first convert the original multi-sensor sequences into more structured degradation representations through signal decomposition, health indicator construction, or trajectory matching. Then, they use recurrent networks or statistical prediction methods to complete the remaining life estimation. These methods can explicitly incorporate degradation trends, stage divisions, and historical evolution patterns into the prediction process.

In terms of trend feature construction, Zhao et al. utilized complete ensemble empirical mode decomposition to decompose the sensor sequence into several intrinsic mode components and residual terms, and constructed candidate trend representations by combining different components; subsequently, they evaluated the candidate trend features using the sensor measurement distribution learned by conditional neural processes and input the selected trend representation into LSTM for RUL prediction [11]. This method combines signal decomposition, trend reconstruction, candidate feature evaluation, and recurrent prediction, emphasizing the role of the overall degradation trend in life prediction.

In terms of health indicators and similarity prediction, Wang et al. first described the evolution process of the engine from a healthy state to a degraded state through fuzzy clustering, and then constructed health indicators based on this. Subsequently, they performed time series decomposition and trend prediction on the sequence of health indicators, and finally estimated the RUL by comparing the similarity between the test trajectory and historical degradation trajectories [12]. This approach incorporates the degradation stage, health indicator evolution, and historical trajectory matching into the prediction process, providing a structured representation for handling nonlinear degradation processes.

Overall, the explicit feature construction method can incorporate information from fields such as signal decomposition, stage priors, and trajectory similarity. However, the trend reconstruction, health indicator generation, time series prediction, and final RUL estimation are usually accomplished through multiple relatively independent steps, making it difficult to form a unified gradient optimization process from the original multi-sensor input to the RUL target. Based on this characteristic, subsequent research has gradually shifted towards using deep networks to directly learn local representations and temporal states from multi-variable sliding windows.

### 2.2. Deep Hybrid Time Series Modeling for RUL Prediction

The deep RUL prediction methods have gradually evolved from single convolution or recurrent structures to multi-stage networks that integrate local feature extraction, long-term dependency modeling, and automatic structure optimization. Babu et al. earlier applied deep convolutional regression to RUL estimation for multivariate degradation sequences [13]; Zheng et al. further utilized LSTM to model the long-term temporal dependencies in industrial degradation sequences [14]. Li et al. constructed a deep convolutional network to directly learn the mapping between degradation representations and RUL from fixed-length multi-sensor windows [15]; Ellefsen et al. adopted a semi-supervised deep architecture, using unlabeled degradation data for pre-training representations, and then completed RUL regression for turbofan engines [16]. Based on this, Li et al. extracted degradation features from different time ranges using multi-scale deep convolution [17], while Mo et al. utilized evolutionary neural architectures to search for and automatically optimize the structure of RUL prediction networks [18].

In the combination route of convolution and LSTM, Elsherif et al. proposed CAELSTM, which uses a convolutional autoencoder to form compressed feature representations, and then completes RUL prediction through LSTM and attention mechanism [19]. Deng and Zhou proposed the CNN-LSTM-Attention model, which uses CNN for front-end feature extraction, then uses LSTM to aggregate time states, and calculates the correlation between sequence representations through standard Query, Key and Value attention [20]. Both of these methods combine convolutional representations and recurrent state modeling, but they adopt different designs in terms of local encoding form, whether including reconstruction processes, and attention calculation methods.

Attention is also used to reorganize the temporal states generated by the recurrent network. Chen et al. first learned sequence representations from the raw sensor data using LSTM, then estimated the importance of different time steps and feature dimensions separately, and fused the automatically learned representations with manually statistically and trend-based features with domain knowledge [21]. This method enables multiple recurrent states to participate in the final prediction with non-uniform weights, while retaining the supplementary information provided by external features. Therefore, the attention mainly acts on the LSTM output and feature fusion stage, which is different from the intermediate context enhancement mechanism located between the convolutional representation and the recurrent network.

Time convolutional networks have also been further combined with GRU or BiGRU. Gong et al. first used random forests to evaluate the contribution of sensors and utilized variational mode decomposition to convert the retained signals into multiple intrinsic mode components; subsequently, they modeled the correlations between these modal representations using an attention mechanism and completed subsequent time feature extraction and bidirectional state aggregation with TCN and BiGRU [22]. Zou and Lin proposed MCA-TCN-GRU, which uses multiple TCN blocks to extract time features, fuses time and channel information from different TCN outputs through multi-channel attention, and then uses GRU to complete RUL prediction [23]. Both of them utilize the complementarity of time convolution and gated recurrent networks, but there are differences in input construction methods, attention objects, convolution output organization, and the direction of recurrent aggregation.

Overall, deep RUL prediction has developed along multiple technical paths, ranging from single CNN or LSTM, semi-supervised and multi-scale representation learning, to convolutional–recurrent hybrid networks and automatic structure optimization. Different models have different emphases in terms of input representation, the way of connecting local and long-term temporal features, the position of attention effect, and the final state aggregation strategy.

### 2.3. Contextual Modeling for Enhanced Attention and the Positioning of This Paper

The role of the attention mechanism in RUL prediction is not the same in terms of the object and insertion position. Existing hybrid models have respectively applied attention to the LSTM output state, the time correlation calculation after the convolution and recurrent representations, the decomposed modal representations, and the fusion of multiple TCN block outputs. These designs indicate that attention can be used not only to re-weight the already generated recurrent states, but also to complete multi-sensor or multi-scale feature fusion before recursive modeling.

Apart from channel concatenation, the graph-structured methods further explicitly model the relationships between sensors. Li et al. utilized hierarchical attention graph convolution to fuse multi-sensor signals, enabling simultaneous learning of sensor associations and degraded representations [24]; Cao et al. further proposed an adaptive dynamic fusion graph neural network, in which multiscale convolutional features are used to construct dynamic sensor relationships, and graph attention together with BiGRU is employed to integrate spatial correlations and temporal dependencies [25]. These methods treat sensors as nodes with structural relationships, providing a modeling approach different from sequence concatenation for the fusion of multi-variable degradation information.

Regarding the joint weighting of temporal positions and sensor contributions, Liu et al. constructed a dual-attention architecture that separately enhances sensor features and temporal representations [26]; Sun et al. integrated an enhanced inverted Transformer with spatiotemporal graph learning to jointly model global multi-sensor dependencies and graph-based spatiotemporal representations [27]; Zhao et al. selectively extracted multi-range degraded features through multi-scale modules and deep self-attention [28]. These methods indicate that the weighted objects in multi-sensor RUL prediction can cover different levels such as channels, time positions, and multi-scale features.

The Transformer architecture further expands the way of modeling global dependencies. Fu et al. proposed PSTFormer, which employs parallel spatial and temporal Transformer branches to capture cross-sensor relationships and temporal dependencies in multi-sensor aero-engine monitoring sequences [29]; another work introduced the degradation trend representation and time features into the Transformer network together [30]. Fan et al. proposed a two-stage hierarchical Transformer, which sequentially learns the attention of the time dimension and the sensor dimension, and extracts representations of different time scales through a hierarchical structure [31]; Ma et al. modeled the sensor relationships using adaptive graph convolution and used the Transformer encoder to extract long-term temporal dependencies [32].

More recently, Fu et al. proposed DKAMFormer, which incorporates domain knowledge into multi-sensor representations and introduces multiscale time-scale-guided self-attention to learn complementary degradation features at different temporal resolutions [33]. Du et al. proposed STF^2^ to extract and fuse spatial and temporal information from multi-sensor sequences in parallel [34]. Qin et al. further developed MSMT-FDA, which combines multi-sensor spatial modeling, multiscale temporal Transformer representations, and frequency-domain attention, and evaluated the model across FD001–FD004 and N-CMAPSS scenarios [35].

The time convolution route has also continuously focused on feature selection, bidirectional context, and attention fusion. Zha and Ye combined sensor feature selection with the improved TCN to enhance the extraction of degradation features for aircraft engines [36]. He et al. combined BiTCN with multi-head attention, forming an interval RUL representation based on forward and backward time convolution, and further applied it to maintenance strategy optimization [37]. Compared with the above methods, MBQG-Net organizes multi-range bidirectional time convolution, window-level QV time attention, and stacked bidirectional GRU in a continuous information flow. Its QV module is located between the convolutional representation and the aggregation of the recurrent state, forming window-level context through Query scoring and Value aggregation, and then enhancing the input of the subsequent gated network in a residual manner. To provide a more structured comparison with representative existing architectures, Table 1 summarizes their main differences in temporal feature extraction, bidirectional modeling, attention formulation and position, and recurrent aggregation.

The comparison indicates that the distinction of MBQG-Net does not arise from the use of convolution, attention, or recurrent modeling alone, but from their ordered organization. Existing RUL prediction methods differ mainly in input representation, bidirectional temporal-context modeling, the objects and positions of attention, and final state aggregation. Whereas recent methods increasingly emphasize explicit cross-sensor dependency modeling and global or multiscale self-attention, MBQG-Net adopts a staged temporal information flow: MS-BiTCN first extracts multi-range bidirectional temporal features, QV attention then performs Query-based temporal weighting and Value aggregation without an independent Key projection, and the stacked BiGRU finally completes bidirectional gated state aggregation. The next section introduces the detailed architecture and training objective of MBQG-Net.

## 3. The Proposed Method

### 3.1. Problem Definition

The prediction of the RUL of an aero-engine is formulated as a nonlinear supervised regression problem that maps a sequence of historical multivariate observations to a continuous RUL value. For the *u*th engine, the multivariate observation vector at the *t*th operating cycle is defined in Equation (1):

(1) $$s_{u,t}={\left[s_{u,t}^{1},s_{u,t}^{2},\ldots ,s_{u,t}^{C}\right]}^{⊤}\in R^{C},$$

where *C* denotes the number of input channels.

Given a sliding window of length *W*, the input sample ending at operating cycle *t* is constructed according to Equation (2):

(2) $$X_{u,t}=\left[s_{u,t-W+1},s_{u,t-W+2},\ldots ,s_{u,t}\right]\in R^{C\times W}.$$

The corresponding true RUL label is denoted by $y_{u,t}$. After all generated windows are re-indexed, the training set is represented as

(3) $$D_{\mathrm{tr}}={\left\{\left(X_{i},y_{i}\right)\right\}}_{i=1}^{N_{\mathrm{tr}}},$$

where $N_{\mathrm{tr}}$ denotes the total number of training-window samples and *i* denotes the window index rather than the engine index. The learning objective is to estimate an end-to-end mapping parameterized by Θ, as defined in Equation (4):

(4) $${\hat{y}}_{i}=f_{\Theta }\left(X_{i}\right),$$

where ${\hat{y}}_{i}$ denotes the predicted RUL of the *i*th input window. For a batch of samples, $X\in R^{B\times C\times W}$, where *B* is the batch size. The specific input-channel configuration and the adopted sliding-window length are detailed in the experimental setup.

**Figure 1:**
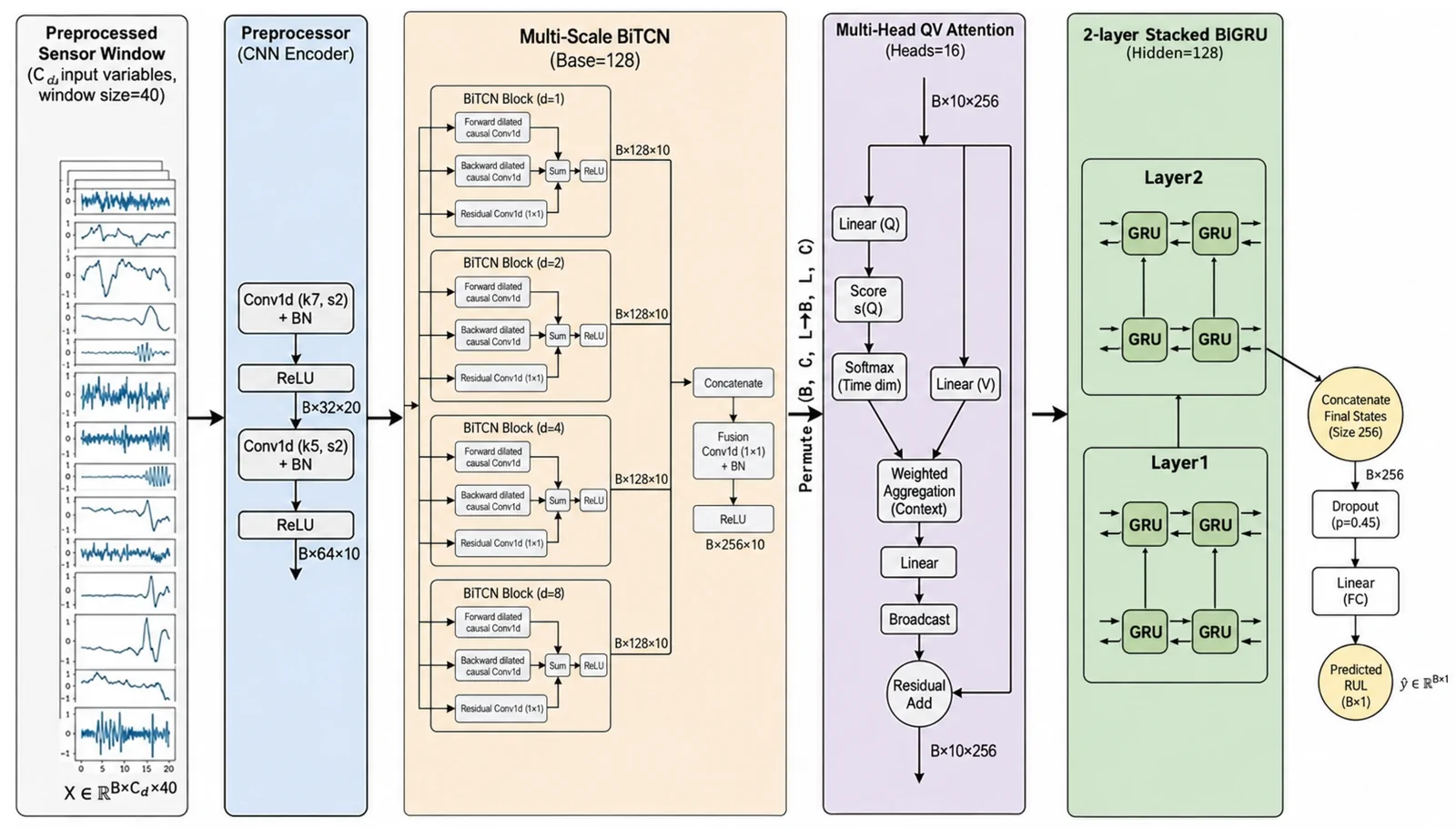
Overall architecture and information flow of MBQG-Net.

To model local variations within a fixed window, multi-range temporal dependencies, and differences in positional contributions, MBQG-Net constructs a staged temporal representation process from multivariate inputs to RUL predictions. Local temporal features are first extracted and compressed by the convolutional encoder, multi-scale bidirectional temporal information is then modeled by MS-BiTCN, and the resulting sequence is further enhanced through QV temporal attention before stacked bidirectional recurrent aggregation. The overall architecture and information flow are illustrated in Figure 1, while the main operations and output tensor formats of individual stages are summarized in Table 2.

Accordingly, the overall forward mapping of MBQG-Net can be compactly expressed by Equation (5a), (5b) and (5c):

(5a) $$\begin{array}{cc} F_{\mathrm{ms}}\; & =E_{\mathrm{ms}\text{-}\mathrm{bitcn}}\left(E_{\mathrm{cnn}}\left(X\right)\right), \end{array}$$

(5b) $$\begin{array}{cc} F_{\mathrm{qv}}\; & =A_{\mathrm{qv}}\left({\mathrm{Permute}}_{\left(0,2,1\right)}\left(F_{\mathrm{ms}}\right)\right), \end{array}$$

(5c) $$\begin{array}{cc} \hat{y}\; & =R\left(G_{\mathrm{bigru}}\left(F_{\mathrm{qv}}\right)\right). \end{array}$$

Here, $E_{\mathrm{cnn}}\left(\cdot \right)$ denotes the local temporal encoder, $E_{\mathrm{ms}\text{-}\mathrm{bitcn}}\left(\cdot \right)$ denotes the multi-scale bidirectional temporal convolution module, $A_{\mathrm{qv}}\left(\cdot \right)$ denotes the multi-head QV temporal attention module, $G_{\mathrm{bigru}}\left(\cdot \right)$ denotes the two-layer stacked bidirectional GRU, and R(·) denotes the final regression head. The operation ${\mathrm{Permute}}_{\left(0,2,1\right)}\left(\cdot \right)$ rearranges the tensor from B×D×T to B×T×D, where *D* and *T* denote the feature dimension and temporal length of the MS-BiTCN output, respectively.

Overall, MBQG-Net forms a continuous temporal representation pipeline that combines local encoding, multi-scale bidirectional temporal modeling, QV-based context enhancement, and stacked bidirectional state aggregation. The following subsections describe the individual components in detail.

### 3.2. Local Time Encoder

The original multivariate window consists of input responses observed over consecutive operating cycles. MBQG-Net first employs two strided one-dimensional convolutional layers to jointly encode local responses from multiple input channels while progressively reducing the temporal length, thereby providing a compact representation for the subsequent convolutional, attention, and recurrent modules.

The convolutional encoding unit used in the local encoder is defined in Equation (6):

(6) $$C_{k,s,p}^{c_{\mathrm{in}}\to c_{\mathrm{out}}}\left(U\right)=\phi \left[\mathrm{BN}\left({Conv1D}_{k,s,p}^{c_{\mathrm{in}}\to c_{\mathrm{out}}}\left(U\right)\right)\right],$$

where *k*, *s*, and *p* denote the convolution kernel size, stride, and padding size, respectively. BN(·) denotes one-dimensional batch normalization, and ϕ(·) denotes the ReLU activation function.

Using this encoding unit, the two-layer local encoding process is expressed by Equation (7a) and (7b):

(7a) $$\begin{array}{cc} F_{\mathrm{cnn}}^{\left(1\right)}\; & =C_{k_{1},s_{1},p_{1}}^{C\to C_{1}}\left(X\right)\in R^{B\times C_{1}\times T_{1}}, \end{array}$$

(7b) $$\begin{array}{cc} F_{\mathrm{cnn}}\; & =C_{k_{2},s_{2},p_{2}}^{C_{1}\to C_{e}}\left(F_{\mathrm{cnn}}^{\left(1\right)}\right)\in R^{B\times C_{e}\times T}. \end{array}$$

Here, $C_{1}$ and $T_{1}$ denote the channel number and temporal length after the first convolutional layer, whereas $C_{e}$ and *T* denote those of the final local-encoder output. The resulting compact representation is then passed to the multi-scale bidirectional temporal convolution module.

### 3.3. Multi-Scale Bidirectional Temporal Convolution

The local time encoder produces $F_{\mathrm{cnn}}\in R^{B\times C_{e}\times T}$. Although the temporal length has been compressed, different dilation rates still provide complementary receptive patterns over the encoded sequence. To integrate temporal information at multiple ranges in both directions, MBQG-Net employs a Multi-scale Bidirectional Temporal Convolution (MS-BiTCN) module.

Let the set of dilation rates be Δ={1,2,4,8}, with a common kernel size K=3. For a dilation rate *d*, the causal dilated convolution at temporal position *t* is defined in Equation (8):

(8) $${\left[{\mathrm{CConv}}_{d}\left(U\right)\right]}_{t}=\sum_{r=0}^{K-1}W_{d,r}U_{t-dr}+b_{d}.$$

Indices outside the valid temporal range are handled by left zero-padding. In the implementation, a temporal padding of $p_{d}=\left(K-1\right)d$ is applied, after which the additional $p_{d}$ positions at the end of the sequence are removed to preserve the original temporal length. This operation is denoted by ${\mathrm{Crop}}_{d}\left(\cdot \right)$.

With the common kernel size K=3, the nominal temporal span of a dilated convolution is $R_{d}=1+\left(K-1\right)d$. Accordingly, the four dilation rates d={1,2,4,8} correspond to receptive spans of 3, 5, 9, and 17 encoded temporal positions, respectively. The dilation rates follow a geometric progression, progressively enlarging the temporal sampling interval while keeping the convolution kernel size fixed. This configuration therefore provides complementary local, intermediate, and wider-range temporal responses through a compact set of parallel branches. The dilation set was adopted as a structured multi-scale configuration rather than selected through an exhaustive search over dilation combinations.

For each dilation rate *d*, the BiTCN branch contains forward, backward, and residual paths, defined by Equation (9a), (9b) and (9c):

(9a) $$\begin{array}{cc} F_{d}^{\mathrm{fwd}}\; & =\phi \left[\mathrm{BN}\left({\mathrm{Crop}}_{d}\left({\mathrm{Conv}}_{d}^{\mathrm{fwd}}\left(F_{\mathrm{cnn}}\right)\right)\right)\right], \end{array}$$

(9b) $$\begin{array}{cc} F_{d}^{\mathrm{bwd}}\; & =\mathrm{Rev}\left\{\phi \left[\mathrm{BN}\left({\mathrm{Crop}}_{d}\left({\mathrm{Conv}}_{d}^{\mathrm{bwd}}\left(\mathrm{Rev}\left(F_{\mathrm{cnn}}\right)\right)\right)\right)\right]\right\}, \end{array}$$

(9c) $$\begin{array}{cc} F_{d}^{\mathrm{res}}\; & =P_{d}^{\mathrm{res}}\left(F_{\mathrm{cnn}}\right). \end{array}$$

Here, Rev(·) denotes reversal along the temporal dimension. The forward path performs causal convolution in the original temporal order, whereas the backward path applies the same sequence of convolution, cropping, batch normalization, and ReLU after temporal reversal and subsequently restores the original order. The residual path employs a 1×1 convolution to align the channel dimension when necessary.

The three responses within each branch are fused element-wise according to Equation (10):

(10) $$B_{d}=\phi \left(F_{d}^{\mathrm{fwd}}+F_{d}^{\mathrm{bwd}}+F_{d}^{\mathrm{res}}\right),\;B_{d}\in R^{B\times C_{b}\times T},$$

where $C_{b}$ denotes the number of output channels of each BiTCN branch.

**Figure 2:**
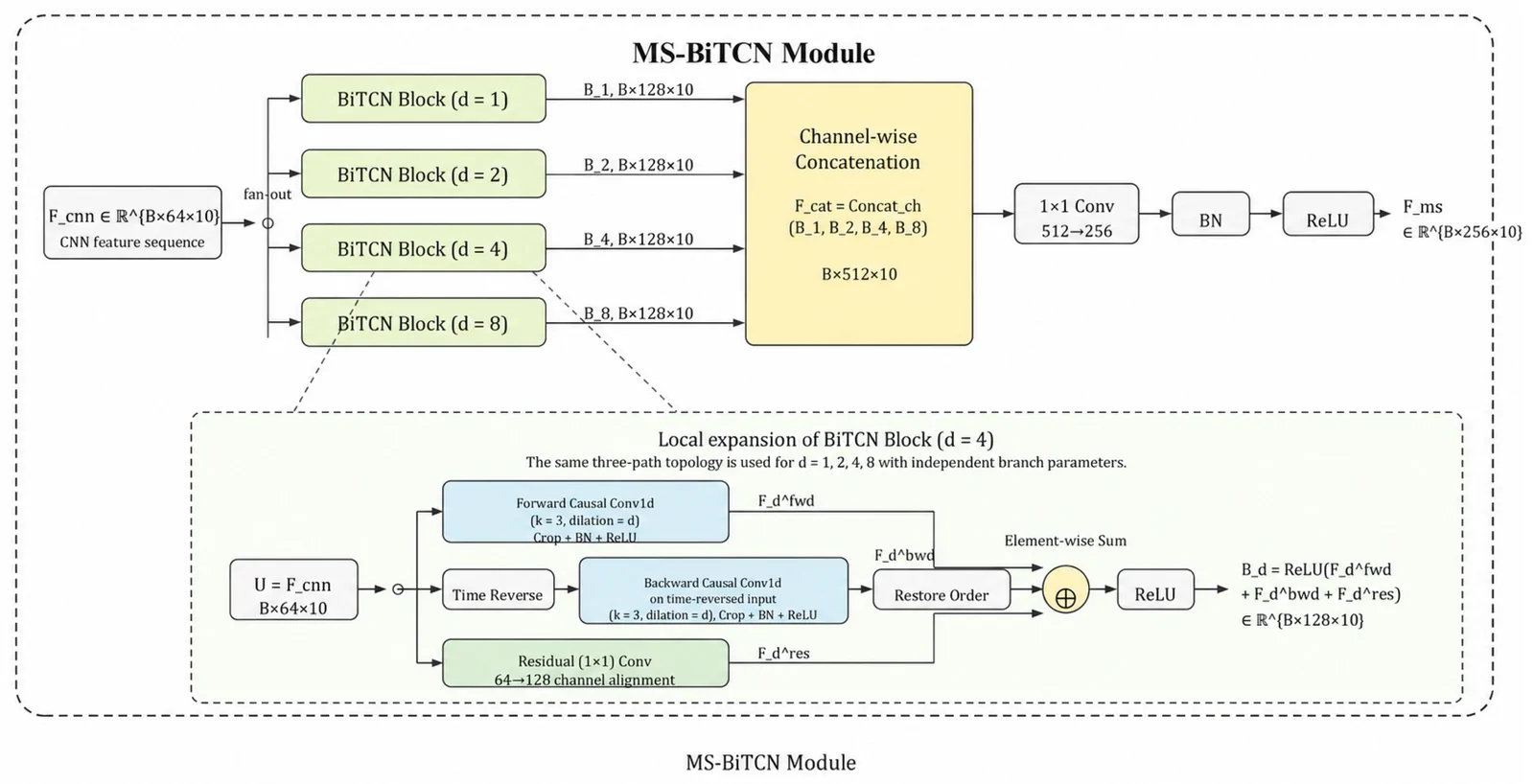
The multi-scale bidirectional temporal convolution structure of the MS-BiTCN module.

The outputs of all dilation branches are subsequently concatenated along the channel dimension and integrated using a 1×1 convolution, batch normalization, and ReLU, as expressed by Equation (11a) and (11b):

(11a) $$\begin{array}{cc} F_{\mathrm{cat}}\; & ={\mathrm{Concat}}_{\mathrm{ch}}\left({\left\{B_{d}\right\}}_{d\in \Delta }\right)\in R^{B\times \left|\Delta \right|C_{b}\times T}, \end{array}$$

(11b) $$\begin{array}{cc} F_{\mathrm{ms}}\; & =\phi \left[\mathrm{BN}\left({\mathrm{Conv}}_{1\times 1}\left(F_{\mathrm{cat}}\right)\right)\right]\in R^{B\times D\times T}, \end{array}$$

where *D* denotes the feature dimension after cross-branch integration. The parallel dilation branches therefore capture complementary temporal patterns over different receptive ranges, while the backward path uses only observations contained within the current historical window and introduces no future information beyond the prediction time. The detailed structure of MS-BiTCN is illustrated in Figure 2.

### 3.4. Multi-Head QV Temporal Attention

After MS-BiTCN processing, the model obtains the temporal feature representation $F_{\mathrm{ms}}\in R^{B\times D\times T}$. Before recurrent state aggregation, it is necessary to further distinguish the relative contributions of different temporal positions within the current observation window. To this end, a multi-head Query–Value (QV) temporal attention mechanism is introduced to perform temporal-position weighting and window-level context aggregation. Unlike conventional QKV self-attention, the proposed mechanism does not explicitly construct pairwise Query–Key similarities between all temporal positions. After permuting the feature and temporal dimensions,

$$Z={\mathrm{Permute}}_{\left(0,2,1\right)}\left(F_{\mathrm{ms}}\right)\in R^{B\times T\times D},$$

and Z is used as the input to the QV attention module.

Let the number of attention heads be *H*, and let the feature dimension of each head be $d_{h}=D/H$. The input sequence is first projected into Query and Value representations, as defined in Equation (12a) and (12b):

(12a) $$\begin{array}{cc} \tilde{Q}\; & =ZW_{Q}+b_{Q}, \end{array}$$

(12b) $$\begin{array}{cc} \tilde{V}\; & =ZW_{V}+b_{V}. \end{array}$$

Here, $W_{Q},W_{V}\in R^{D\times D}$ and $b_{Q},b_{V}\in R^{D}$. After reorganizing the projected features into the multi-head form, the resulting representations satisfy

$$Q,V\in R^{B\times H\times T\times d_{h}}.$$

For the *b*th sample, the *h*th attention head, and the *t*th temporal position, a shared scalar scoring function is first applied to the Query representation, followed by Softmax normalization along the temporal dimension. The corresponding operations are given by Equation (13a) and (13b):

(13a) $$\begin{array}{cc} e_{b,h,t}\; & =w_{s}^{⊤}q_{b,h,t}+b_{s}, \end{array}$$

(13b) $$\begin{array}{cc} \alpha_{b,h,t}\; & =\frac{\exp\left(e_{b,h,t}\right)}{\sum_{\tau =1}^{T}\exp\left(e_{b,h,\tau }\right)}. \end{array}$$

The parameters $w_{s}\in R^{d_{h}}$ and $b_{s}\in R$ define the temporal-position scoring function and are shared across attention heads. This shared scoring mechanism applies a common temporal-importance criterion to all heads, while representation diversity is retained through the different Query and Value subspaces induced by the multi-head partition. It also avoids introducing an independent scoring function for each attention head.

Using the temporal weights obtained from Equation (13b), each attention head aggregates its corresponding Value representations into a head-level window context:

(14) $$c_{b,h}=\sum_{t=1}^{T}\alpha_{b,h,t}v_{b,h,t}\in R^{d_{h}}.$$

The context vectors from all heads are then organized and vectorized into a *D*-dimensional window-level context representation, as expressed in Equation (15a) and (15b):

(15a) $$\begin{array}{cc} M_{b}\; & =\left[\begin{array}{c} c_{b,1}^{⊤}\\ c_{b,2}^{⊤}\\ \vdots \\ c_{b,H}^{⊤} \end{array}\right]\in R^{H\times d_{h}}, \end{array}$$

(15b) $$\begin{array}{cc} c_{b}\; & =\mathrm{vec}\left(M_{b}\right)\in R^{D}. \end{array}$$

Here, vec(·) denotes column-wise vectorization. Since $D=Hd_{h}$, the resulting $c_{b}$ forms the window-level context vector of the *b*th sample. This ordering is consistent with the implementation, in which the head and within-head feature dimensions are rearranged before being flattened into a *D*-dimensional representation. After the window-level context vectors of all samples in a batch are organized as $C\in R^{B\times D}$, an output affine mapping is first applied. The mapped context is then repeated along the temporal dimension and injected into the original temporal representation through a residual connection. This process is compactly expressed by Equation (16a) and (16b):

(16a) $$\begin{array}{cc} C_{o}\; & =CW_{o}+b_{o},\;C_{o}\in R^{B\times D}, \end{array}$$

(16b) $$\begin{array}{cc} F_{\mathrm{qv}}\; & =Z+{\mathrm{Repeat}}_{T}\left(C_{o}\right)\in R^{B\times T\times D}. \end{array}$$

**Figure 3:**
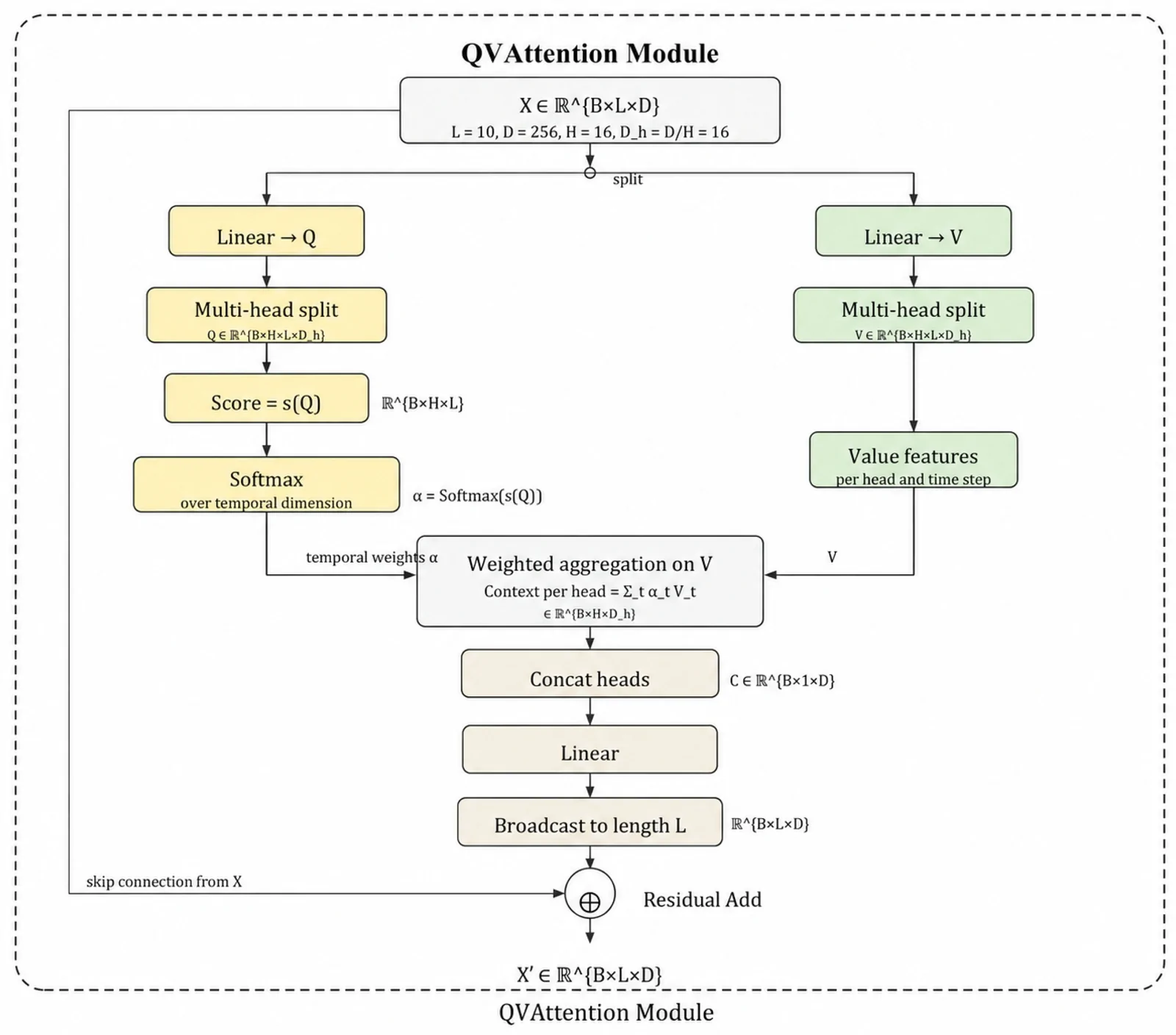
The process of multi-head QV temporal attention and window-level context residual injection.

Here, $W_{o}\in R^{D\times D}$ and $b_{o}\in R^{D}$. Therefore, the proposed QV temporal attention directly derives temporal-position weights from Query representations and aggregates the corresponding Value representations into a window-level context, rather than constructing pairwise Query–Key similarities. The resulting context is mapped, broadcast along the temporal dimension, and residually injected into the original sequence to obtain the enhanced representation $F_{\mathrm{qv}}$. The complete QV temporal attention and window-level context residual-injection process is illustrated in Figure 3.

### 3.5. Stacked Bidirectional GRU State Aggregation

After QV temporal attention, the model obtains the enhanced sequence $F_{\mathrm{qv}}\in R^{B\times T\times D}$. To further aggregate bidirectional temporal states within the fixed observation window, a two-layer stacked bidirectional GRU is employed to recursively model the enhanced temporal representation. The final hidden states from both directions of the top layer are subsequently used to construct a window-level representation.

For the input $u_{t}$ at temporal position *t* in a given direction and the previous hidden state $h_{t-1}$, the GRU state update is defined by Equation (17a), (17b), (17c) and (17d):

(17a) $$\begin{array}{cc} r_{t}\; & =\sigma \left(W_{ir}u_{t}+b_{ir}+W_{hr}h_{t-1}+b_{hr}\right), \end{array}$$

(17b) $$\begin{array}{cc} z_{t}\; & =\sigma \left(W_{iz}u_{t}+b_{iz}+W_{hz}h_{t-1}+b_{hz}\right), \end{array}$$

(17c) $$\begin{array}{cc} {\tilde{h}}_{t}\; & =\tanh\left[W_{in}u_{t}+b_{in}+r_{t}⊙\left(W_{hn}h_{t-1}+b_{hn}\right)\right], \end{array}$$

(17d) $$\begin{array}{cc} h_{t}\; & =\left(1-z_{t}\right)⊙{\tilde{h}}_{t}+z_{t}⊙h_{t-1}. \end{array}$$

Here, $r_{t}$ and $z_{t}$ denote the reset and update gates, respectively, ${\tilde{h}}_{t}$ denotes the candidate hidden state, σ(·) denotes the Sigmoid function, and ⊙ denotes element-wise multiplication.

For the *l*th BiGRU layer, the forward and backward hidden states are recursively updated in the original and reversed temporal directions, respectively. Their state transitions are expressed by Equation (18a) and (18b):

(18a) $$\begin{array}{cc} {\vec{h}}_{t}^{\left(l\right)}\; & ={\mathrm{GRU}}_{\mathrm{fwd}}^{\left(l\right)}\left(u_{t}^{\left(l\right)},{\vec{h}}_{t-1}^{\left(l\right)}\right), \end{array}$$

(18b) $$\begin{array}{cc} {\overset{\leftarrow }{h}}_{t}^{\left(l\right)}\; & ={\mathrm{GRU}}_{\mathrm{bwd}}^{\left(l\right)}\left(u_{t}^{\left(l\right)},{\overset{\leftarrow }{h}}_{t+1}^{\left(l\right)}\right). \end{array}$$

The first BiGRU layer directly receives the QV-enhanced sequence, such that $u_{t}^{\left(1\right)}=F_{\mathrm{qv}}\left[:,t,:\right]$. The forward and backward hidden states produced by the first layer are concatenated along the feature dimension and passed through inter-layer Dropout before being used as input to the second BiGRU layer:

(19) $$u_{t}^{\left(2\right)}=\mathrm{Dropout}\left(\left[{\vec{h}}_{t}^{\left(1\right)};{\overset{\leftarrow }{h}}_{t}^{\left(1\right)}\right],p_{\mathrm{gru}}\right).$$

The notation [·;·] denotes concatenation along the feature dimension, and $p_{\mathrm{gru}}$ denotes the inter-layer Dropout probability.

Let the hidden dimension in each direction be $d_{g}$, and let the number of stacked BiGRU layers be $L_{g}$. After the top-layer bidirectional state updates are completed, the final forward hidden state ${\vec{h}}_{T}^{\left(L_{g}\right)}$ and the final backward hidden state ${\overset{\leftarrow }{h}}_{1}^{\left(L_{g}\right)}$ are concatenated to form the window-level representation. The resulting vector is then passed through an output Dropout layer and a linear regression layer to obtain the final RUL prediction, as defined by Equation (20a) and (20b):

(20a) $$\begin{array}{cc} g\; & =\left[{\vec{h}}_{T}^{\left(L_{g}\right)};{\overset{\leftarrow }{h}}_{1}^{\left(L_{g}\right)}\right]\in R^{B\times 2d_{g}}, \end{array}$$

(20b) $$\begin{array}{cc} \hat{y}\; & =\mathrm{Dropout}\left(g,p_{\mathrm{out}}\right)W_{r}+b_{r}. \end{array}$$

**Figure 4:**
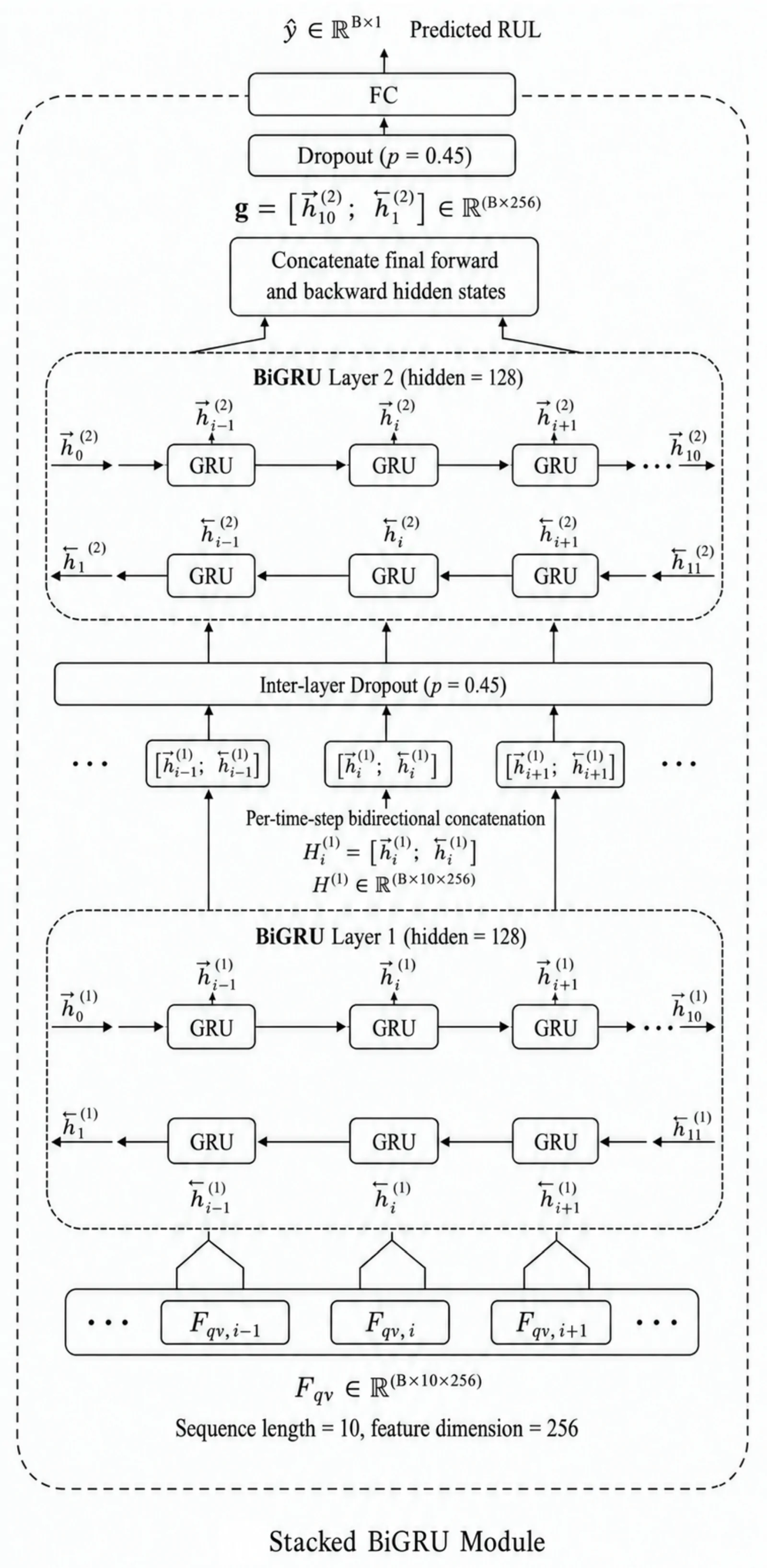
Stacked bidirectional GRU aggregation process.

Here, $W_{r}\in R^{2d_{g}\times 1}$ and $b_{r}\in R$ denote the weight and bias of the final linear regression layer, respectively, and $\hat{y}\in R^{B\times 1}$. In the configuration adopted in this study, $L_{g}=2$ and $d_{g}=128$. Therefore, concatenating the two final directional states of the top BiGRU layer yields a $2d_{g}=256$-dimensional window-level representation for the final RUL regression. The stacked bidirectional GRU aggregation process is illustrated in Figure 4.

### 3.6. Asymmetric Weighted MSE for RUL Overestimation Risk

Conventional MSE assigns the same penalty to positive and negative prediction errors with the same absolute magnitude. However, RUL overestimation and underestimation have different implications for maintenance decision-making. For the *i*th sample, the prediction error is defined as $e_{i}={\hat{y}}_{i}-y_{i}$: $e_{i}>0$ indicates that the predicted RUL is higher than the true RUL, whereas $e_{i}<0$ indicates RUL underestimation. The NASA Score applies a faster-growing exponential penalty to RUL overestimation. To align the training objective with this asymmetric error direction, a direction-dependent weighted squared error is adopted. The weighting function and batch-wise training loss are defined by Equation (21a) and (21b):

(21a) $$\begin{array}{cc} w(e_{i})\; & =\left\{\begin{array}{cc} \lambda_{\mathrm{under}}, & e_{i}\leq 0,\\ \lambda_{\mathrm{over}}, & e_{i}>0, \end{array}\right. \end{array}$$

(21b) $$\begin{array}{cc} L_{\mathrm{asym}}^{\left(B\right)}\; & =\frac{1}{B}\sum_{i=1}^{B}w\left(e_{i}\right)e_{i}^{2}. \end{array}$$

In this study, $\lambda_{\mathrm{under}}=1$ and $\lambda_{\mathrm{over}}=2$, so that a larger training weight is assigned to RUL overestimation errors. The 2:1 ratio is used as a fixed heuristic asymmetric weighting scheme to place greater emphasis on overestimation errors; it is not intended to represent a calibrated 2:1 ratio between the actual maintenance costs associated with overestimation and underestimation.

The proposed training objective therefore employs a direction-dependent weighted quadratic penalty and is consistent with the NASA Score in terms of the direction of asymmetric penalization. Their mathematical forms nevertheless remain different: the proposed loss uses direction-dependent weighted squared errors, whereas the NASA Score uses direction-dependent exponential penalties. The detailed definition of the NASA Score, together with the other evaluation metrics, is provided in the evaluation-metrics section.

## 4. Experimental Setup and Evaluation

### 4.1. Dataset Description

To verify the effectiveness of the proposed MBQG-Net in the task of predicting the RUL of aircraft engines, this paper conducts experiments using the commercial modular aerospace propulsion system simulation dataset (Commercial Modular Aero-Propulsion System Simulation, C-MAPSS) released by NASA Prognostics Data Repository [38]. This dataset generates multi-engine time series with multiple sensors from healthy operation to gradual degradation through simulation of turbofan engine operation, and is widely used in the research of RUL prediction for aircraft engines.

The C-MAPSS includes four sub-datasets: FD001, FD002, FD003, and FD004. Each original record consists of the engine number, operating cycle, three operating condition variables, and 21 sensor measurement values. The four sub-datasets differ in terms of the number of engines, operating conditions, and fault modes. Their basic information is shown in Table 3. The number of training and test records in the table represents the number of cycle-level observation records in the original file, not the number of model input samples obtained after sliding window processing.

Each engine in the training set has a complete degradation trajectory from the initial operating state until the failure moment, so the actual RUL of its final operating cycle is 0. In contrast, the engine trajectories in the test set are truncated before the failure occurs. The test files only provide sensor records up to the current observation cycle; NASA provides the actual remaining useful life of each test engine corresponding to the final observation cycle for the final model evaluation.

This paper conducts experiments on all four C-MAPSS sub-datasets. FD001 and FD003 contain a single operating condition, whereas FD002 and FD004 contain multiple operating conditions. In terms of fault modes, FD001 and FD002 contain one fault mode, while FD003 and FD004 contain two fault modes. These combinations provide complementary evaluation scenarios for examining the prediction performance of MBQG-Net under changes in both operating-condition variability and degradation complexity.

The symbols, physical meanings and units of the 21 sensor variables in C-MAPSS are listed in Table 4. This table also indicates the 14 sensors actually used by MBQG-Net, so as to directly correspond with the input tensors defined in the method section.

### 4.2. Data Preprocessing and Sample Construction

The original C-MAPSS records cannot be directly used as fixed-length inputs to the neural network. Therefore, the preprocessing pipeline sequentially performs engine-level data partitioning, input-variable selection, normalization based only on training-set statistics, piecewise-linear RUL label construction, and sliding-window sample generation. The overall procedure follows the order of first partitioning engines, then fitting preprocessing parameters using only the training split, and finally generating window samples. This design prevents highly correlated windows from the same engine from being simultaneously assigned to the training and validation sets.

#### 4.2.1. Engine-Level Partition and Input Variable Configuration

For each C-MAPSS sub-dataset, the available training engines are divided into training and validation subsets at a fixed ratio of approximately 8:2. The partition is performed at the engine level so that all samples generated from the same engine remain in the same subset. This avoids information leakage caused by highly correlated windows originating from the same degradation trajectory. The exact numbers of training and validation engines for each sub-dataset are reported in Table 5.

To quantitatively characterize the degradation-related temporal variation of the sensor signals, the within-condition temporal variation of all 21 sensor channels was evaluated using the training partition only, with the within-condition standard deviation adopted as a unified measure. For the single-condition subsets FD001 and FD003, this measure reduces to the standard deviation calculated directly over the training records. For the multi-condition subsets FD002 and FD004, the standard deviation was calculated separately within each operating condition and then pooled according to the number of training records in each condition, thereby avoiding the inclusion of between-condition baseline shifts as degradation-related temporal variation. Specifically, the pooled within-condition standard deviation of sensor *j* is defined as

(22) $$\sigma_{j,\mathrm{within}}=\sqrt{\sum_{c}\frac{n_{c}}{N}\sigma_{j,c}^{2}},$$

where $n_{c}$ denotes the number of training records under operating condition *c*, *N* is the total number of training records, and $\sigma_{j,c}$ denotes the standard deviation of sensor *j* within condition *c*.

The statistical results show that, among all 21 sensor channels, $s_{1}$, $s_{5}$, $s_{18}$, and $s_{19}$ exhibit zero within-condition variation across all four C-MAPSS subsets. Sensors $s_{6}$, $s_{10}$, and $s_{16}$ likewise exhibit very limited within-condition temporal variation, with the largest pooled within-condition standard deviations across the four subsets being 0.0189, 0.0050, and 0.0016, respectively, and the corresponding maximum within-condition ranges being 0.16, 0.05, and 0.01. Collectively, these seven channels form the group with the most limited temporal variation among the sensor variables and are therefore treated as low-information variables and excluded from the model input.

This sensor configuration is also consistent with recent C-MAPSS studies in which the same seven channels are removed because they provide limited degradation-related information [39].

After excluding these channels, the retained sensor set is defined in Equation (23):

(23) $$S=\{s_{2},s_{3},s_{4},s_{7},s_{8},s_{9},s_{11},s_{12},s_{13},s_{14},s_{15},s_{17},s_{20},s_{21}\}.$$

Equation (23) therefore retains 14 sensor variables. The same sensor subset is used consistently across all four C-MAPSS sub-datasets to maintain a common sensor representation. For the single-condition subsets FD001 and FD003, these 14 sensor variables constitute the complete model input. For the multi-condition subsets FD002 and FD004, the three operating-setting variables are additionally retained as input variables and are also used to identify the operating conditions required for condition-aware standardization. Consequently, the model input dimension is 14 for FD001/FD003 and 17 for FD002/FD004.

#### 4.2.2. Training-Set-Based Normalization

Different input variables have different physical quantities and numerical ranges. To prevent variables with larger numerical scales from having a disproportionate influence on gradient updates, all normalization parameters are estimated exclusively from the training-engine records.

For the single-condition subsets FD001 and FD003, Z-score normalization is applied to the 14 retained sensor channels using statistics calculated from the complete training split. For the multi-condition subsets FD002 and FD004, condition-aware standardization is adopted. The three operating-setting variables are rounded to two decimal places and jointly used to identify the operating condition associated with each observation. For each operating condition, independent normalization statistics are estimated from the training split for all 17 input variables, including the three operating-setting variables and the 14 retained sensor channels. Accordingly, the normalization procedure is summarized in Equation (24):

(24) $$\begin{array}{cc} {\tilde{x}}_{u,t}^{\left(c\right)} & =\left\{\begin{array}{cc} \frac{x_{u,t}^{\left(c\right)}-\mu_{c}^{\mathrm{train}}}{\sigma_{c}^{\mathrm{train}}},\; & \mathrm{FD}001/\mathrm{FD}003,\\ \frac{x_{u,t}^{\left(c\right)}-\mu_{k,c}^{\mathrm{train}}}{\sigma_{k,c}^{\mathrm{train}}},\; & \mathrm{FD}002/\mathrm{FD}004,\;k=k(u,t). \end{array}\right. \end{array}$$

Here, $x_{u,t}^{\left(c\right)}$ denotes the value of the *c*th input variable of engine *u* at cycle *t*, while $\mu_{c}^{\mathrm{train}}$ and $\sigma_{c}^{\mathrm{train}}$ denote the mean and standard deviation calculated from the training records for the corresponding input variable. The same training-set statistics are applied to the validation and official test sets without recalculation, thereby preventing validation- or test-set information from entering the preprocessing stage.

For FD002 and FD004, k(u,t) denotes the operating condition associated with the observation at cycle *t*, and $\mu_{k,c}^{\mathrm{train}}$ and $\sigma_{k,c}^{\mathrm{train}}$ denote the condition-specific mean and standard deviation of the *c*th input variable, estimated only from the training records belonging to condition *k*. The same condition-specific statistics are applied to the validation and official test sets. If an operating condition not observed in the training split is encountered, a global StandardScaler fitted on all training records and all retained input variables is used as a fallback.

**Figure 5:**
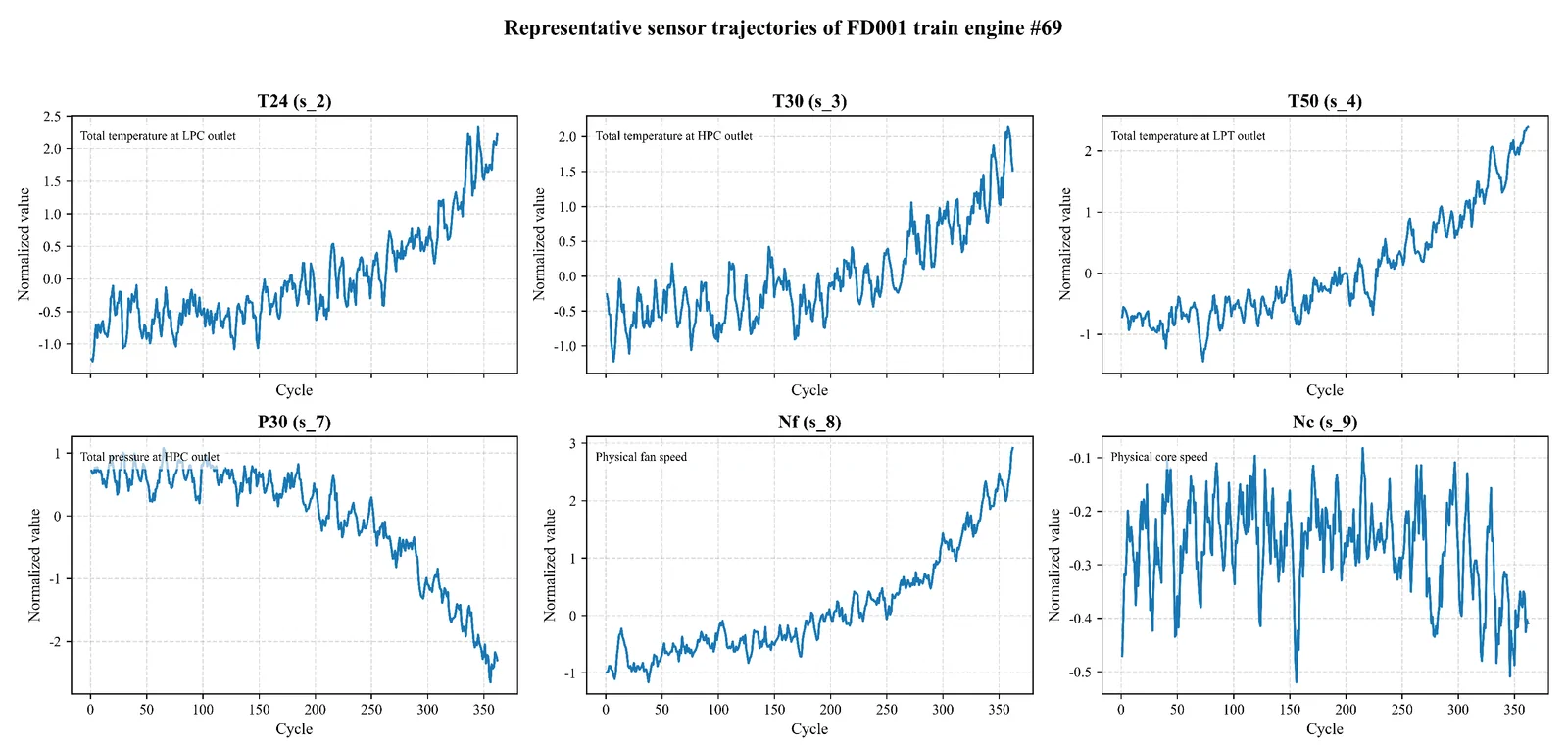
The standardized sensor trajectory of a representative engine from the FD001 training set.

To illustrate the temporal behavior of the standardized signals, Figure 5 shows the trajectories of six representative sensors from one FD001 training engine.

#### 4.2.3. Piecewise-Linear RUL Labeling

For the *u*th engine in the training set, let its complete lifespan consist of $T_{u}$ operating cycles. The uncapped RUL at operating cycle *t* is defined in Equation (25):

(25) $$r_{u,t}=T_{u}-t.$$

To avoid excessively large RUL values during the early healthy stage, this study adopts a piecewise-linear supervision label with an upper bound of $R_{\max}=125$, as defined in Equation (26):

(26) $$y_{u,t}=\min\left(T_{u}-t,R_{\max}\right),\;R_{\max}=125.$$

**Figure 6:**
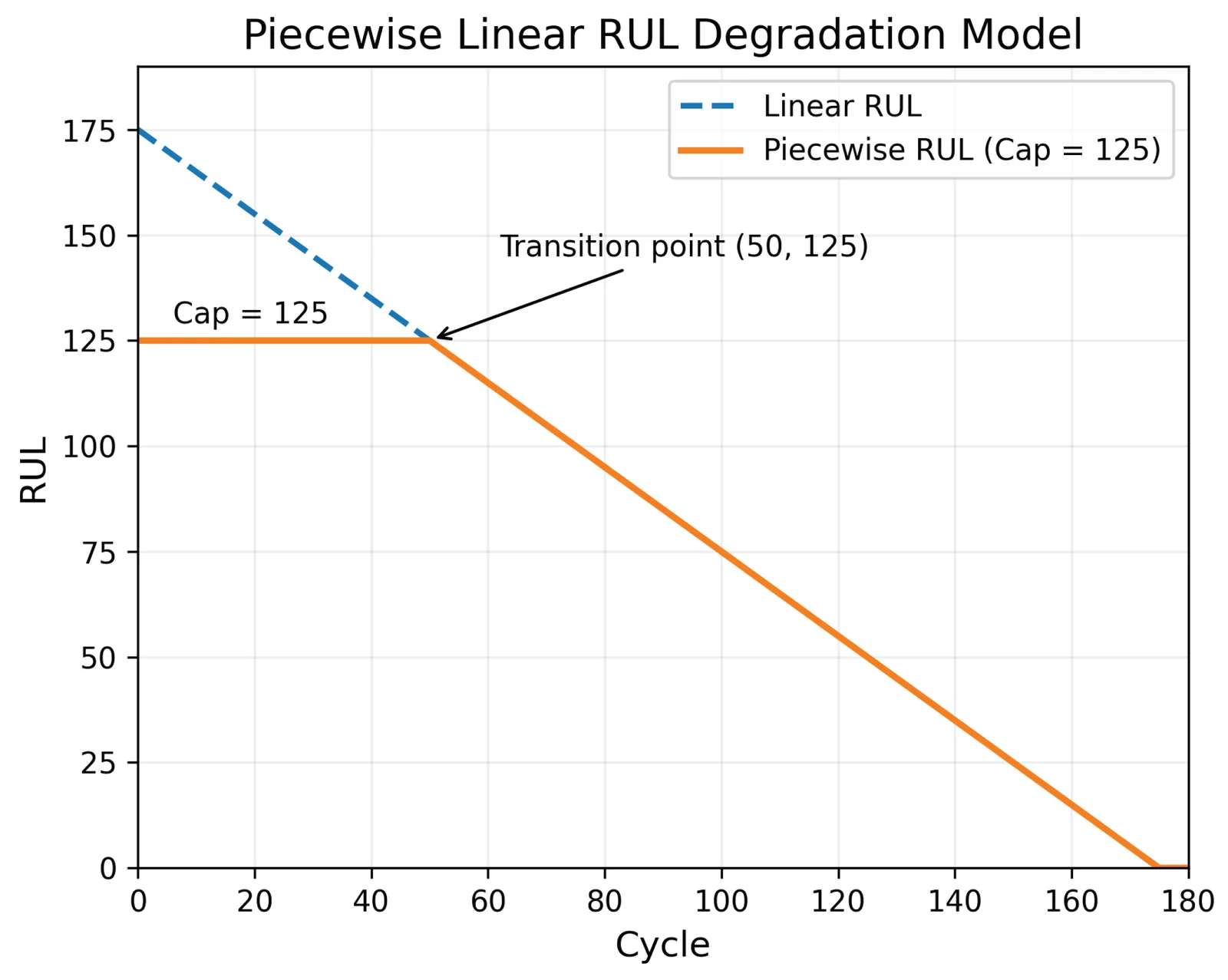
Piecewise-linear RUL label construction with an upper limit of 125.

When the uncapped RUL in Equation (25) exceeds $R_{\max}$, the supervision label remains fixed at 125. After the engine enters the later degradation stage, the label decreases linearly with the operating cycle and reaches 0 at failure. The relationship between the uncapped and piecewise-linear RUL labels is illustrated in Figure 6.

The official C-MAPSS test trajectories are truncated before failure and therefore do not contain complete degradation lifetimes. NASA provides the true remaining useful life of each test engine at its final observation cycle for final evaluation. For consistency with the training-label definition in Equation (26), the official test RUL values are capped using the same upper bound $R_{\max}=125$ before evaluation.

#### 4.2.4. Sliding Window Sample Construction

To convert engine trajectories with different lifetimes into fixed-size neural-network inputs, a sliding window of length W=40 is adopted with a sliding step size of 1. Let $C_{d}$ denote the number of input variables for sub-dataset *d*, where $C_{d}=14$ for FD001/FD003 and $C_{d}=17$ for FD002/FD004. For the *u*th engine, the window ending at operating cycle *t* is constructed according to Equation (27):

(27) $$X_{u,t}=\left[{\tilde{x}}_{u,t-W+1},{\tilde{x}}_{u,t-W+2},\ldots ,{\tilde{x}}_{u,t}\right]\in R^{C_{d}\times W},\;W=40.$$

**Figure 7:**
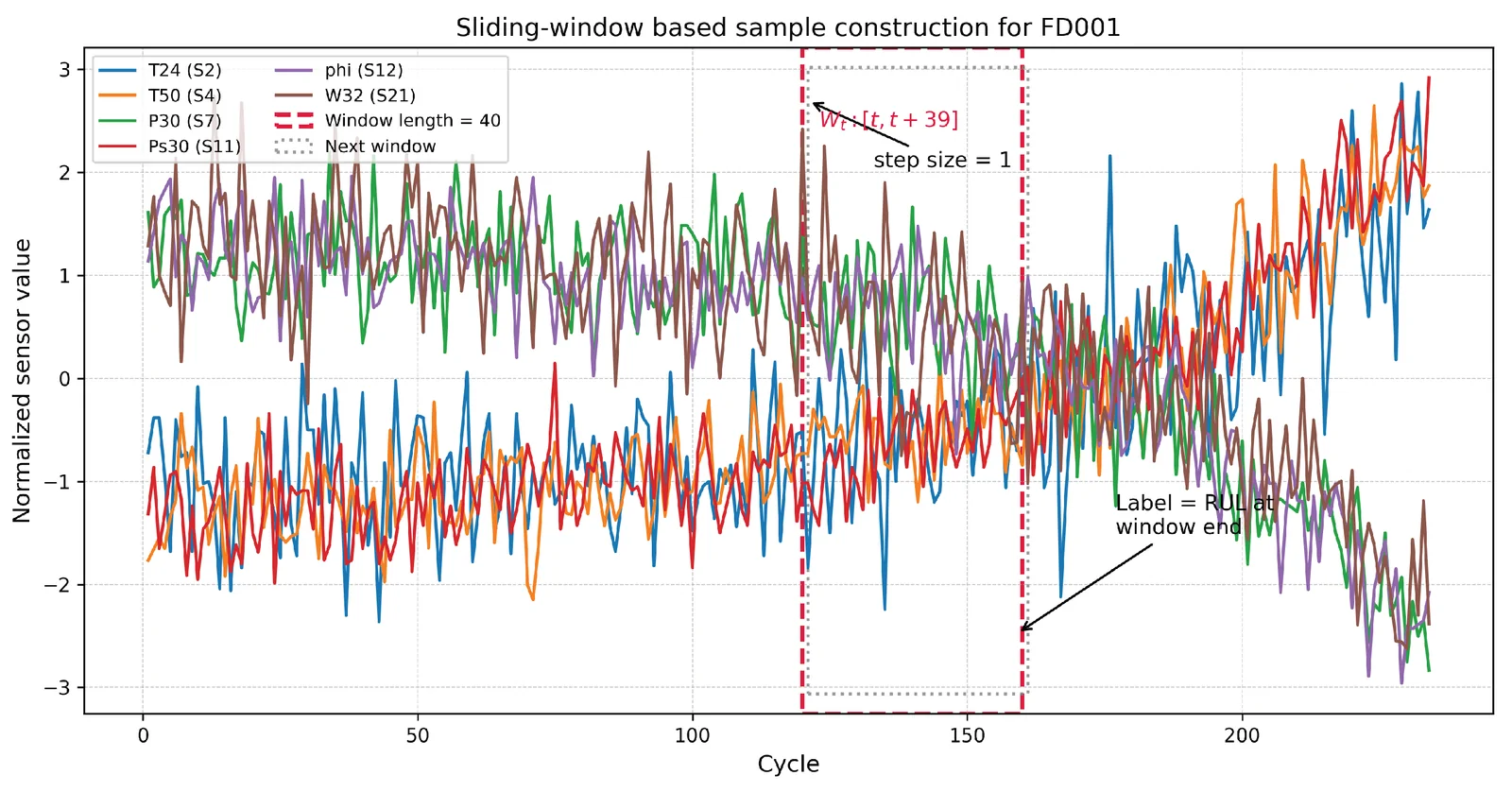
The process of constructing a sliding window sample with a length of 40.

The corresponding regression target is the RUL label at the final cycle of the window, that is, $X_{u,t}\to y_{u,t}$. The sliding-window construction process is illustrated in Figure 7.

For a training or validation engine containing $L_{u}$ operating cycles, the number of available window samples is given by Equation (28):

(28) $$N_{u}=L_{u}-W+1,\;L_{u}\geq W.$$

During the official testing phase, the final W=40 observation cycles of each engine are used to construct one test input, from which the RUL at the final observation time is predicted. Each test engine therefore contributes one official test sample. If the length of a test trajectory is shorter than 40 cycles, zero padding is applied to the beginning of the sequence to maintain a consistent input size. The numbers of engines and generated window samples after engine-level partitioning and sliding-window construction are summarized in Table 5.

### 4.3. Evaluation Metrics

To evaluate the RUL prediction performance, this study adopts Root Mean Square Error (RMSE), Mean Absolute Error (MAE), the coefficient of determination ($R^{2}$), and NASA Score. Let the official test set contain *N* samples, with $y_{i}$ and ${\hat{y}}_{i}$ denoting the true and predicted RUL of the *i*th sample, respectively. Consistent with Section 3.6, the prediction error is defined as $e_{i}={\hat{y}}_{i}-y_{i}$, where $e_{i}>0$ indicates RUL overestimation and $e_{i}<0$ indicates RUL underestimation. Under the official C-MAPSS test protocol, each test engine contributes one final evaluation sample.

RMSE and MAE quantify the overall magnitude of prediction errors and are defined in Equation (29a) and (29b):

(29a) $$\begin{array}{cc} \mathrm{RMSE}\; & =\sqrt{\frac{1}{N}\sum_{i=1}^{N}{\left({\hat{y}}_{i}-y_{i}\right)}^{2}}, \end{array}$$

(29b) $$\begin{array}{cc} \mathrm{MAE}\; & =\frac{1}{N}\sum_{i=1}^{N}\left|{\hat{y}}_{i}-y_{i}\right|. \end{array}$$

RMSE assigns greater influence to large prediction deviations because of the squared-error term, whereas MAE measures the average absolute deviation between predicted and true RUL values. The coefficient of determination evaluates the agreement between the predicted and true RUL values and is calculated according to Equation (30):

(30) $$R^{2}=1-\frac{\sum_{i=1}^{N}{\left(y_{i}-{\hat{y}}_{i}\right)}^{2}}{\sum_{i=1}^{N}{\left(y_{i}-\bar{y}\right)}^{2}},$$

where $\bar{y}=N^{-1}\sum_{i=1}^{N}y_{i}$ denotes the mean true RUL over the official test samples. A higher $R^{2}$ indicates stronger agreement between the predicted and true RUL values.

NASA Score further evaluates the direction-sensitive prediction error. Its total score and single-sample penalty function are defined in Equation (31a) and (31b):

(31a) $$\begin{array}{cc} S\; & =\sum_{i=1}^{N}\phi \left(e_{i}\right), \end{array}$$

(31b) $$\begin{array}{cc} \phi (e_{i})\; & =\left\{\begin{array}{cc} \exp\left(-\frac{e_{i}}{13}\right)-1,\; & e_{i}<0,\\ \exp\left(\frac{e_{i}}{10}\right)-1,\; & e_{i}\geq 0. \end{array}\right. \end{array}$$

**Figure 8:**
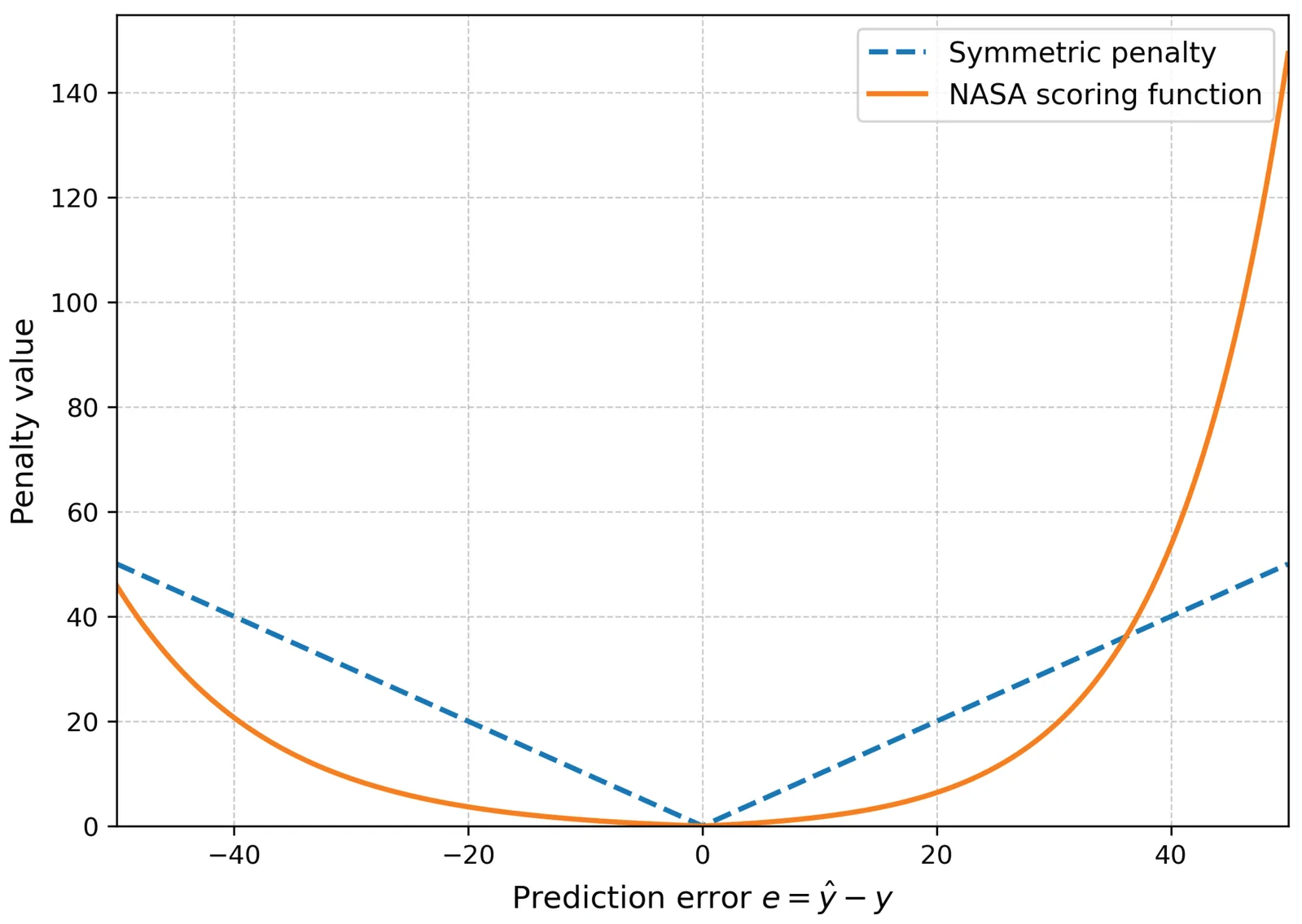
Comparison of the single-sample absolute error response with the NASA asymmetric scoring response.

Because the positive-error branch in Equation (31b) uses the smaller exponential denominator, the penalty increases more rapidly for $e_{i}>0$, thereby placing greater emphasis on RUL overestimation. Figure 8 illustrates the difference between the conventional absolute-error response and the direction-sensitive NASA penalty. Lower RMSE, MAE, and NASA Score values indicate better prediction performance, whereas a higher $R^{2}$ is preferred.

### 4.4. Experimental Setup

For the main comparative experiments, all four C-MAPSS sub-datasets adopted the same core network architecture and training configuration listed in Table 6, unless otherwise specified in the controlled sensitivity, ablation, and attention-comparison experiments. Following the preprocessing protocol in Section 4.2, the seven low-information sensor channels ($s_{1},s_{5},s_{6},s_{10},s_{16},s_{18},s_{19}$) were excluded, and the remaining 14 sensor channels were retained for all four sub-datasets. For FD002 and FD004, the three operating-setting variables were additionally retained as input variables and were also used to identify the operating conditions required for condition-aware standardization. The sliding-window length and step size were set to 40 and 1, respectively, while the upper bound of the piecewise-linear RUL label was fixed at 125. The model was trained for 120 epochs on each sub-dataset using a batch size of 512. The RUL upper bound of 125 follows the commonly adopted piecewise-linear labeling strategy for C-MAPSS, which limits the early-life RUL plateau and facilitates comparison with previous studies.

The optimization was performed using AdamW with a maximum learning rate of $5\times 10^{-4}$ and a weight-decay coefficient of $8\times 10^{-4}$. The learning rate was scheduled using OneCycleLR with pct_start = 0.45. Both the dropout between layers in the stacked BiGRU and the dropout before the regression head were set to 0.45. The training objective was the asymmetric weighted MSE defined in Section 3.6, with an overestimation weight of 2 and an underestimation weight of 1. The effects of key hyperparameter settings on model performance were examined through controlled one-factor sensitivity analyses. Specifically, six window lengths, W∈{20,30,35,40,45,50}, four weight-decay coefficients, $\lambda_{\mathrm{wd}}\in \left\{0,4\times 10^{-4},8\times 10^{-4},1.6\times 10^{-3}\right\}$, and three asymmetric-loss weighting ratios, $\lambda_{\mathrm{over}}:\lambda_{\mathrm{under}}\in \left\{1:1,2:1,3:1\right\}$, were evaluated on FD004 while the remaining network architecture and training settings were kept fixed. The corresponding results are reported in Section 4.5, treated as fixed configurations rather than being subjected to exhaustive hyperparameter tuning.

The selection of the model checkpoint was performed using the validation set. For the *i*th validation sample, the prediction error is defined as

(32) $$e_{i}^{\mathrm{val}}={\hat{y}}_{i}^{\mathrm{val}}-y_{i}^{\mathrm{val}}.$$

The mean NASA penalty and the hybrid validation criterion are defined in Equation (33a) and (33b):

(33a) $$\begin{array}{cc} {\bar{S}}_{\mathrm{val}}\; & =\frac{1}{N_{\mathrm{val}}}\sum_{i=1}^{N_{\mathrm{val}}}\phi \left(e_{i}^{\mathrm{val}}\right), \end{array}$$

(33b) $$\begin{array}{cc} J_{\mathrm{val}}\; & ={\bar{S}}_{\mathrm{val}}+0.15\;{\mathrm{RMSE}}_{\mathrm{val}}. \end{array}$$

The function ϕ(·) follows the NASA direction-sensitive penalty defined in Equation (31b). Using the mean NASA penalty rather than its sum prevents the number of validation samples from directly affecting the numerical scale of the criterion. The coefficient 0.15 in Equation (33b) was fixed throughout all experiments and was not separately optimized; it retains RMSE as a secondary conventional-error component while the mean NASA penalty provides the direction-sensitive component of checkpoint evaluation. All main training runs used a fixed budget of 120 epochs without an additional patience-based early-stopping rule. The checkpoint corresponding to the minimum $J_{\mathrm{val}}$ over the training process was retained for final evaluation on the official C-MAPSS test set.

All experiments were implemented using Python 3.10.14, NumPy 1.26.4, and PyTorch 2.1.2 built with CUDA 11.8. The experiments were conducted on a system equipped with an AMD Ryzen 9 7945HX processor, 16 GB of system memory, and an NVIDIA GeForce RTX 4060 Laptop GPU with 8 GB of GPU memory. Automatic mixed precision (AMP) was enabled during training. To assess sensitivity to random initialization, MBQG-Net was additionally evaluated over five independent runs on each sub-dataset while keeping the data partition, architecture, and training settings unchanged; the corresponding mean and standard deviation are reported separately. The main experimental configuration is summarized in Table 6.

### 4.5. Comparative Experiments and Result Analysis

To comprehensively evaluate MBQG-Net, a three-level comparison framework is adopted, including conventional regression methods, representative deep temporal models, and structure-oriented baselines. For a fair comparison, all methods follow the same engine-level training–validation split, input-feature configuration, normalization protocol, piecewise-linear RUL labeling, sliding-window length, and official NASA test protocol. The official test set is used only for final performance reporting and is not involved in model selection.

The first level includes SVR and XGBoost as conventional nonlinear regression baselines. SVR represents kernel-based nonlinear regression, whereas XGBoost represents tree-based ensemble learning. These methods provide non-deep baselines for assessing the benefit of explicit temporal representation learning in MBQG-Net.

The second level includes PCA-LSTM, Attention-LSTM, and CNN-LSTM-Attention as representative deep temporal baselines. PCA-LSTM represents dimensionality-reduction-assisted recurrent modeling, Attention-LSTM represents attention-enhanced recurrent temporal modeling, and CNN-LSTM-Attention represents convolutional–recurrent–attention hybrid modeling. Together, these methods provide representative deep temporal references for assessing the overall prediction capability of MBQG-Net.

The third level consists of Fast-BiTCN, TCN-BiGRU, TCN-QVAttention, and TCN-Attention-GRU as structure-oriented baselines. Rather than serving as additional general-purpose reference models, these baselines are used to examine specific structural factors that are directly related to MBQG-Net, including bidirectional temporal convolution, bidirectional recurrent aggregation, QV temporal weighting, and conventional attention-based temporal aggregation. This design enables a more targeted comparison of the structural combination adopted in MBQG-Net.

The RMSE, MAE, $R^{2}$, and NASA Score obtained by all compared methods on the official test sets of FD001–FD004 are summarized in Table 7. Since the four C-MAPSS subsets differ in operating conditions and fault-mode complexity, the following analysis discusses the results from a cross-scenario perspective.

FD001 and FD003 both represent single-condition scenarios, while differing in fault-mode complexity: FD001 contains one fault mode and FD003 contains two. They therefore provide a useful basis for examining whether the comparative performance of MBQG-Net is maintained as fault-mode complexity increases without additional operating-condition variation.

A consistent performance pattern is observed across the two single-condition subsets: MBQG-Net achieves the best results for all four evaluation metrics on both FD001 and FD003. In particular, the NASA Score decreases from the second-best 290.1598 to 203.5562 on FD001 and from 307.9764 to 217.6334 on FD003, while the corresponding RMSE improvements are 5.19% and 11.57%, respectively. Thus, the comprehensive advantage observed on FD001 remains evident on FD003 despite the increase from one to two fault modes, indicating stable conventional regression and direction-sensitive prediction performance under the single-condition setting.

FD002 and FD004, in contrast, both contain six operating conditions and therefore introduce additional variation associated with changing operating regimes. FD002 contains one fault mode, whereas FD004 contains two, making FD004 more heterogeneous in terms of both operating-condition and fault-mode variation.

The multi-condition subsets exhibit a less uniform comparative pattern. On FD002, MBQG-Net achieves the best MAE and the numerically lowest NASA Score, whereas TCN-BiGRU obtains slightly better RMSE and $R^{2}$. The NASA Scores of MBQG-Net and TCN-Attention-GRU are 952.2441 and 952.7822, respectively, showing that the advantage on this metric is marginal. On FD004, the comparative advantage becomes more pronounced: MBQG-Net achieves the best RMSE, $R^{2}$, and NASA Score, although its MAE is not the lowest. In particular, the NASA Score decreases from the second-best 1297.7732 to 930.9088, corresponding to a 28.27% reduction. These results show that the strength and metric profile of the advantage vary between the two multi-condition subsets rather than remaining uniform.

Considering all four C-MAPSS subsets together, two main patterns emerge. MBQG-Net shows comprehensive four-metric superiority on the single-condition FD001 and FD003 subsets, whereas its conventional regression advantage becomes more metric-dependent under the multi-condition FD002 and FD004 settings. In contrast, NASA Score is the only metric for which MBQG-Net obtains the lowest value on all four subsets, although the margin on FD002 is very small. Overall, the results demonstrate strong performance across the evaluated degradation scenarios without implying uniform superiority on every conventional regression metric.

**Figure 9:**
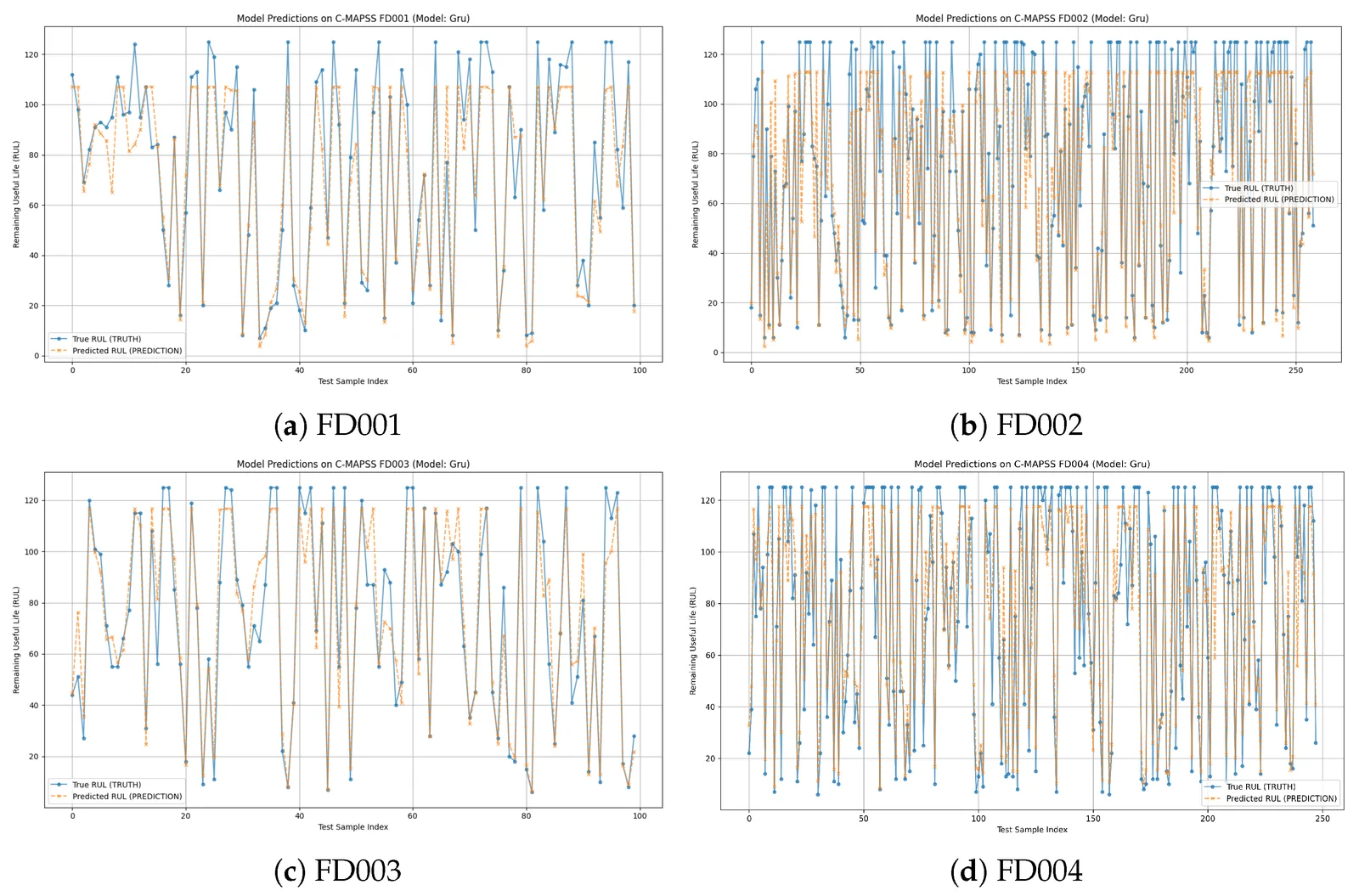
RUL prediction results of MBQG-Net on the official test sets: (**a**) FD001, (**b**) FD002, (**c**) FD003, and (**d**) FD004.

Figure 9 provides a qualitative comparison between the predicted and ground-truth RUL values of MBQG-Net on the four official test sets. These prediction curves complement the quantitative results in Table 7 by illustrating the model behavior under different combinations of operating conditions and fault modes.

#### 4.5.1. Statistical Robustness Analysis

To further evaluate the sensitivity of MBQG-Net to random initialization, five independent runs with different random initializations were conducted on each of the four C-MAPSS subsets while keeping the data partition, network architecture, and training hyperparameters unchanged. The mean and standard deviation of RMSE, MAE, $R^{2}$, and NASA Score are reported in Table 8.

Overall, MBQG-Net exhibits relatively stable conventional regression performance across different random initializations. The standard deviation of $R^{2}$ remains below 0.01 on all four subsets, the maximum standard deviation of RMSE is 0.5422, and the standard deviations of MAE range from 0.3659 to 0.5121. These results indicate that although random initialization introduces a certain degree of performance variation, its influence on the overall regression accuracy of MBQG-Net remains relatively limited. In comparison, the NASA Score exhibits greater variability, particularly on FD004, where the result is 1006.1312±119.6150. Because the NASA scoring function is direction-sensitive and applies an exponential penalty to prediction errors, changes in the direction or magnitude of errors for a relatively small number of samples can lead to more pronounced variations in the final score. Therefore, the repeated-run results show that MBQG-Net maintains relatively stable conventional regression performance under different random initializations, while the NASA Score is more sensitive than RMSE, MAE, and $R^{2}$ to local variations in prediction errors.

#### 4.5.2. Hyperparameter Sensitivity Analysis

To further investigate the influence of key hyperparameter settings on the performance of MBQG-Net, sensitivity analyses of the sliding-window length, weight-decay coefficient, and asymmetric-loss weighting ratio were conducted on FD004 (Table 9). FD004 contains six operating conditions and two fault modes and therefore represents a heterogeneous operating and degradation scenario among the four C-MAPSS subsets. All other data-processing procedures, network configurations, and training settings were kept unchanged while only the hyperparameter under investigation was varied. This design enables a focused evaluation of performance variations under different configurations while avoiding repetitive sensitivity experiments across all four subsets.

For the window-size analysis, the prediction performance first improved and then deteriorated as the temporal context was progressively extended. When the window length increased from 20 to 40, RMSE decreased from 20.2143 to 14.1937 and MAE decreased from 15.4952 to 10.8627, corresponding to reductions of approximately 29.78% and 29.90%, respectively. Meanwhile, $R^{2}$ increased from 0.7788 to 0.8909, and the NASA Score decreased from 2859.0315 to 930.9088, corresponding to a reduction of approximately 67.44%. At W=40, all four evaluation metrics achieved their best values among the tested window lengths. Further increasing the window length to 45 or 50 resulted in different degrees of performance deterioration. These results suggest that an excessively short input window may provide insufficient degradation-history information, whereas extending the temporal range beyond an appropriate scale does not necessarily yield additional predictive benefit. Among the evaluated configurations, W=40 therefore provides an appropriate temporal context for the present model and experimental setting.

For the weight-decay analysis, the regularization strength also produced a clear influence on prediction performance. Compared with the configuration without weight decay, setting the coefficient to $8\times 10^{-4}$ reduced RMSE from 15.7581 to 14.1937 and MAE from 11.7530 to 10.8627, corresponding to improvements of approximately 9.93% and 7.58%, respectively. At the same time, $R^{2}$ increased from 0.8656 to 0.8909, while the NASA Score decreased from 1398.5339 to 930.9088, representing a reduction of approximately 33.44%. The settings of $4\times 10^{-4}$ and $1.6\times 10^{-3}$ also generally outperformed the unregularized configuration but did not achieve the overall performance obtained with $8\times 10^{-4}$. These results indicate that moderate weight decay is beneficial to the generalization performance of MBQG-Net, whereas either weaker or stronger regularization did not produce better results under the present experimental conditions.

For the asymmetric-loss weighting ratio, the results show a clear benefit from introducing additional emphasis on RUL overestimation, but also indicate that further increasing this emphasis does not continuously improve prediction performance. Compared with the symmetric 1:1 setting, the adopted 2:1 configuration reduced RMSE, MAE, and NASA Score by approximately 8.72%, 5.59%, and 28.47%, respectively, while increasing $R^{2}$ from 0.8691 to 0.8909. This indicates that explicitly assigning a larger penalty to RUL overestimation is beneficial not only for the direction-sensitive NASA metric but also for conventional regression accuracy. When the overestimation weight was further increased to 3:1, the model still outperformed the symmetric setting, but its overall performance was inferior to that obtained with 2:1. Relative to 3:1, the 2:1 configuration further reduced RMSE, MAE, and NASA Score by approximately 6.32%, 3.22%, and 6.35%, respectively, and achieved a higher $R^{2}$. These results indicate that the adopted 2:1 ratio provides a more appropriate balance between direction-sensitive overestimation control and overall regression accuracy among the examined weighting configurations, thereby providing quantitative support for its use in the proposed training objective.

### 4.6. Ablation Experiment

To verify the actual roles of different components in MBQG-Net, controlled ablation experiments were conducted on FD001 and FD004. FD001 represents the relatively simple single-operating-condition and single-fault-mode setting, whereas FD004 contains multiple operating conditions and two fault modes. Using these two subsets therefore allows the contributions of different components to be examined under both relatively simple and more heterogeneous degradation scenarios without redundantly repeating the same ablation study on all four subsets. All model variants used the same data partitioning, input configuration, sliding-window length, number of training epochs, optimizer, learning-rate schedule, and validation-based checkpoint-selection strategy, with only the component or training objective under investigation being modified.

Specifically, *w/o MS-BiTCN* replaced the multi-scale bidirectional temporal convolution module with a 1×1 projection to preserve the feature dimensions of the subsequent modules while removing multi-dilation bidirectional temporal modeling. *w/o QV Attention* bypassed the QV attention module and directly forwarded the MS-BiTCN output to the BiGRU. *Single-layer BiGRU* reduced the stacked bidirectional GRU from two layers to one layer. *Standard MSE* retained the complete network architecture but replaced the asymmetric weighted MSE with standard MSE. The results of the complete MBQG-Net are identical to those reported in the main comparative experiments. The ablation results are summarized in Table 10.

On FD001, removing MS-BiTCN causes the largest structural degradation, increasing RMSE by 22.21% and NASA Score by 94.01%. Reducing the stacked BiGRU to a single layer increases RMSE by 6.82%, whereas removing QV Attention increases RMSE by only 1.60%. These results indicate that multi-scale bidirectional temporal feature extraction provides the most pronounced structural contribution in the relatively simple single-condition and single-fault-mode setting.

The structural components become more influential on the more heterogeneous FD004 subset. Removing MS-BiTCN, QV Attention, and reducing the stacked BiGRU to a single layer increase RMSE by 34.38%, 50.78%, and 27.30%, respectively. In particular, removing QV Attention also increases MAE by 57.15% and reduces $R^{2}$ from 0.8909 to 0.7521, showing that QV-based temporal weighting contributes more substantially when multiple operating conditions and fault modes are jointly present.

The loss-function ablation shows a different pattern. On FD001, replacing the asymmetric weighted MSE with standard MSE increases RMSE, MAE, and NASA Score by 8.91%, 11.27%, and 23.48%, respectively. On FD004, RMSE and MAE increase by 8.77% and 4.57%, whereas NASA Score increases by 79.06%, from 930.9088 to 1666.9019. The substantially larger change in NASA Score indicates that the asymmetric objective provides a particularly pronounced contribution to direction-sensitive error control. Overall, the ablation results confirm complementary contributions from MS-BiTCN, QV temporal attention, stacked BiGRU, and the asymmetric weighted MSE to the complete MBQG-Net.

#### Comparison with Conventional QKV Attention

To further compare the proposed QV temporal attention with conventional multi-head QKV attention, a controlled experiment was conducted on FD004 (Table 11). The QKV module employed scaled dot-product attention with Query, Key, and Value projections, while its internal attention dimension was set to 192 with 16 heads to approximately match the parameter budget of QV. All other network components, data-processing procedures, training settings, and checkpoint-selection criteria were kept unchanged.

The complete QV and QKV models differ by only 47 trainable parameters and have nearly identical model-state sizes. Under this matched-capacity setting, QV achieves an RMSE of 14.1937, an MAE of 10.8627, an $R^{2}$ of 0.8909, and a NASA Score of 930.9088, compared with 15.0615, 11.4965, 0.8772, and 1091.4073 for QKV. Relative to QKV, QV reduces RMSE and MAE by approximately 5.76% and 5.51%, increases $R^{2}$ by 0.0137, and reduces NASA Score by approximately 14.71%. QV also records a slightly shorter training time under the same experimental configuration. Overall, QV provides better conventional regression and direction-sensitive prediction performance under essentially the same model scale.

## 5. Conclusions

This paper proposes MBQG-Net, a Multi-Scale Bidirectional Query-Guided Gated Network for aero-engine remaining useful life prediction. The model organizes local temporal encoding, multi-scale bidirectional temporal feature extraction, window-level context enhancement, and bidirectional recurrent state aggregation within a continuous information flow. MS-BiTCN employs parallel bidirectional temporal convolution branches with different dilation rates to extract complementary degradation information over different temporal ranges. QV temporal attention is applied before recurrent state aggregation, where Query-based temporal scoring and Value aggregation are used to form a window-level context, which is then residually injected to enhance the input representation of the subsequent BiGRU. In addition, an asymmetric weighted MSE imposes stronger constraints on RUL overestimation errors, allowing the training process to account for both conventional regression accuracy and direction-sensitive error control.

Under a unified experimental protocol, MBQG-Net was systematically evaluated on all four C-MAPSS sub-datasets, FD001–FD004. The experimental results show that the model achieves strong overall regression performance on FD001 and FD003 while maintaining competitive conventional regression accuracy on the more complex FD002 and FD004 subsets. Meanwhile, MBQG-Net achieves the lowest NASA Score among the compared methods on all four sub-datasets. In particular, the NASA Score on FD004 is 930.9088, representing a 28.27% reduction relative to the second-best result. Ablation experiments verify the complementary contributions of MS-BiTCN, QV temporal attention, stacked BiGRU, and the asymmetric training objective to the complete model. Further hyperparameter sensitivity analyses show that, within the investigated range, a window length of W=40 provides a reasonable prediction performance, while moderate weight decay is more beneficial to model training than the unregularized setting. Moreover, under an approximately matched parameter budget, QV and conventional QKV attention achieve comparable conventional regression accuracy, whereas QV obtains a lower NASA Score, indicating that its performance is not simply attributable to additional model capacity. Five independent runs further show that the conventional regression performance remains generally stable under different random initializations. Overall, MBQG-Net achieves a favorable balance between conventional regression accuracy and direction-sensitive error control across different operating-condition and fault-mode settings through the coordinated organization of multi-scale bidirectional temporal representation, window-level temporal enhancement, bidirectional gated state aggregation, and a direction-sensitive training objective. Future work will further investigate the generalization capability of the model under complex operating conditions and cross-domain scenarios, and examine the effects of sensor configuration, RUL-label settings, and asymmetric loss weighting on prediction behavior. On this basis, adaptive direction-sensitive optimization, domain adaptation, and uncertainty estimation can be further explored to improve the applicability and reliability of the model in practical predictive-maintenance tasks.

## Figures and Tables

**Table 1:** Structural comparison between representative RUL prediction architectures and MBQG-Net.

Method	Temporal Feature Extraction	Bidirectional Modeling	Attention Formulation and Position	Recurrent Aggregation
CNN-LSTM-Attention [20]	CNN	No	Standard QKV attention applied to sequence representations after convolutional and recurrent modeling	LSTM
Attention-based LSTM [21]	LSTM-based temporal representation	No	Temporal- and feature-level attention applied to recurrent representations and feature fusion	LSTM
Attention TCN-BiGRU [22]	TCN with decomposed multi-sensor representations	Yes	Attention applied to the decomposed feature representations before subsequent temporal and recurrent aggregation	BiGRU
MCA-TCN-GRU [23]	Multiple TCN blocks	No	Multi-channel attention used to fuse temporal and channel information from different TCN outputs	GRU
**MBQG-Net**	**CNN + parallel multi-scale BiTCN**	**Yes**	**QV temporal attention between MS-BiTCN and BiGRU; query scoring and Value aggregation without an independent Key projection**	**Stacked BiGRU**

**Table 2:** The information flow and tensor form of each stage of MBQG-Net.

Stage	Main Operations	Output Tensor Format
Input window	Multivariate sliding window	$X\in R^{B\times C\times W}$
First-layer convolution	Local temporal encoding and downsampling	$F_{\mathrm{cnn}}^{\left(1\right)}\in R^{B\times C_{1}\times T_{1}}$
Second-layer convolution	Further local encoding and temporal compression	$F_{\mathrm{cnn}}\in R^{B\times C_{e}\times T}$
BiTCN branch	Forward convolution, backward convolution, and residual fusion	$B_{d}\in R^{B\times C_{b}\times T}$
Multi-branch concatenation	Concatenation of all dilation branches along the channel dimension	$F_{\mathrm{cat}}\in R^{B\times \left|\Delta \right|C_{b}\times T}$
Cross-branch integration	1×1 convolution and feature fusion	$F_{\mathrm{ms}}\in R^{B\times D\times T}$
Dimension transformation	Exchange of the feature and temporal dimensions	$Z\in R^{B\times T\times D}$
QV temporal attention	Temporal-position weighting, context aggregation, and residual injection	$F_{\mathrm{qv}}\in R^{B\times T\times D}$
Stacked BiGRU	Concatenation of the top-layer forward and backward final hidden states	$g\in R^{B\times 2d_{g}}$
Regression head	Dropout and linear mapping	$\hat{y}\in R^{B\times 1}$

**Table 3:** Basic information of the four C-MAPSS sub-datasets used in this paper.

Item	FD001	FD002	FD003	FD004
The number of training engines	**100**	**260**	**100**	**249**
The number of testing engines	**100**	**259**	**100**	**248**
The number of operating conditions	**1**	**6**	**1**	**6**
The number of failure modes	**1**	**1**	**2**	**2**
The number of original training cycle records	**20,631**	**53,759**	**24,720**	**61,249**
The number of original test cycle records	**13,096**	**33,991**	**16,596**	**41,214**

**Table 4:** C-MAPSS sensor variables and their usage in this paper.

Sensor	Symbol	Description	Unit	Used
$s_{1}$	T2	Total temperature at fan inlet	°R	No
$s_{2}$	T24	Total temperature at LPC outlet	°R	Yes
$s_{3}$	T30	Total temperature at HPC outlet	°R	Yes
$s_{4}$	T50	Total temperature at LPT outlet	°R	Yes
$s_{5}$	P2	Pressure at fan inlet	psia	No
$s_{6}$	P15	Total pressure in bypass duct	psia	No
$s_{7}$	P30	Total pressure at HPC outlet	psia	Yes
$s_{8}$	Nf	Physical fan speed	rpm	Yes
$s_{9}$	Nc	Physical core speed	rpm	Yes
$s_{10}$	epr	Engine pressure ratio	–	No
$s_{11}$	Ps30	Static pressure at HPC outlet	psia	Yes
$s_{12}$	phi	Ratio of fuel flow to Ps30	pps/psi	Yes
$s_{13}$	NRf	Corrected fan speed	rpm	Yes
$s_{14}$	NRc	Corrected core speed	rpm	Yes
$s_{15}$	BPR	Bypass ratio	–	Yes
$s_{16}$	farB	Burner fuel–air ratio	–	No
$s_{17}$	htBleed	Bleed enthalpy	–	Yes
$s_{18}$	Nf_dmd	Demanded fan speed	rpm	No
$s_{19}$	PCNfR_dmd	Demanded corrected fan speed	rpm	No
$s_{20}$	W31	HPT coolant bleed	lbm/s	Yes
$s_{21}$	W32	LPT coolant bleed	lbm/s	Yes

**Table 5:** The engine-level partition and the number of samples after sliding window construction.

Dataset	Training Engines	Validation Engines	Training Windows	Validation Windows	Official Test Samples
FD001	80	20	13,541	3190	100
FD002	208	52	34,833	8786	259
FD003	80	20	17,089	3731	100
FD004	200	49	41,054	10,484	248

**Table 6:** The main experimental settings of MBQG-Net.

Hyperparameter	Value
*Data configuration*	
Dataset	FD001, FD002, FD003, FD004
Training–validation split	Approximately 8:2 (engine level)
Additional operating-setting variables	0 (FD001/FD003), 3 (FD002/FD004)
Window size/sliding step	40/1
RUL upper bound	125
*Network architecture*	
CNN output channels	32, 64
CNN kernel sizes/strides	7, 5/2, 2
Dilation rates	1, 2, 4, 8
Attention heads	16
Stacked BiGRU layers	2
Hidden size per direction	128
Dropout rate	0.45
*Optimization configuration*	
Epochs/batch size	120/512
Optimizer	AdamW
Maximum learning rate	$5\times 10^{-4}$
Weight decay	$8\times 10^{-4}$
Learning-rate scheduler	OneCycleLR
pct_start	0.45
Loss function	Asymmetric weighted MSE
Overestimation/underestimation weight	2.0/1.0
Mixed precision	Enabled

**Table 7:** Comparison of RUL prediction performance on the four C-MAPSS subsets.

Method	RMSE ↓	MAE ↓	$R^{2}$↑	NASA Score ↓
**FD001**				
SVR	20.2463	14.7598	0.7447	1273.5399
XGBoost	19.5018	14.4781	0.7632	1271.6580
PCA-LSTM	14.4827	10.6435	0.8694	307.4829
Attention-LSTM	15.9481	12.0586	0.8416	577.5002
CNN-LSTM-Attention	13.2537	9.6582	0.8906	290.1598
Fast-BiTCN	20.7787	15.0617	0.7311	1208.9673
TCN-BiGRU	14.5423	10.4874	0.8683	292.8471
TCN-QVAttention	17.8752	12.8041	0.8010	878.3451
TCN-Attention-GRU	14.1556	10.4102	0.8752	414.0797
**MBQG-Net**	**12.5659**	**9.3952**	**0.9017**	**203.5562**
**FD002**				
SVR	17.9420	12.4836	0.8254	1673.3076
XGBoost	17.8383	12.9758	0.8274	1519.3586
PCA-LSTM	15.5454	11.9522	0.8689	1113.4746
Attention-LSTM	15.6226	12.0165	0.8676	1235.1410
CNN-LSTM-Attention	14.8561	11.2802	0.8803	1297.5551
Fast-BiTCN	21.8083	15.8792	0.7421	3109.4702
TCN-BiGRU	**14.6292**	11.4081	**0.8839**	958.2595
TCN-QVAttention	20.6097	15.2309	0.7696	2525.5107
TCN-Attention-GRU	15.0910	11.6932	0.8765	952.7822
**MBQG-Net**	14.8706	**11.2713**	0.8801	**952.2441**
**FD003**				
SVR	16.0524	11.4231	0.8320	732.7079
XGBoost	15.5406	10.8042	0.8426	862.5107
PCA-LSTM	14.5898	10.0378	0.8612	832.9505
Attention-LSTM	13.0347	9.5580	0.8892	522.9247
CNN-LSTM-Attention	14.7822	10.1221	0.8576	801.0189
Fast-BiTCN	18.7883	13.0178	0.7699	875.1484
TCN-BiGRU	13.2931	8.8895	0.8848	354.3935
TCN-QVAttention	19.1946	14.4788	0.7598	987.5861
TCN-Attention-GRU	12.9946	9.9229	0.8899	307.9764
**MBQG-Net**	**11.4908**	**8.2964**	**0.9139**	**217.6334**
**FD004**				
SVR	17.9999	12.8685	0.8246	2493.6173
XGBoost	17.8049	13.0795	0.8284	1543.2422
PCA-LSTM	15.9565	11.0454	0.8622	1543.7676
Attention-LSTM	14.9391	10.8391	0.8792	1297.7732
CNN-LSTM-Attention	15.8266	11.6576	0.8644	1310.1365
Fast-BiTCN	20.1565	15.1660	0.7801	2163.5239
TCN-BiGRU	15.2541	10.4803	0.8740	1373.0191
TCN-QVAttention	20.0108	15.0205	0.7832	2196.2651
TCN-Attention-GRU	14.8758	**9.8566**	0.8802	1316.2382
**MBQG-Net**	**14.1937**	10.8627	**0.8909**	**930.9088**

Note: Lower RMSE, MAE, and NASA Score values indicate better performance, whereas higher $R^{2}$ values are better. Bold and underline indicate the best and second-best results within each sub-dataset, respectively.

**Table 8:** Statistical robustness of MBQG-Net over five independent runs on the four C-MAPSS subsets.

Dataset	RMSE	MAE	$R^{2}$	NASA Score
FD001	13.1284±0.5422	9.8887±0.4475	0.8925±0.0088	224.9866±20.3181
FD002	15.1744±0.0693	11.7693±0.3659	0.8751±0.0011	852.9186±42.0029
FD003	11.8379±0.3795	8.5446±0.4629	0.9086±0.0059	227.0621±9.3492
FD004	15.0088±0.2895	11.2420±0.5121	0.8780±0.0048	1006.1312±119.6150

Note: Results are reported as mean ± standard deviation over five independent runs with different random initializations.

**Table 9:** Hyperparameter sensitivity analysis of MBQG-Net on FD004.

(a) Window-size sensitivity				
**Window size**	**RMSE ↓**	**MAE ↓**	$R^{2}$↑	**NASA Score ↓**
20	20.2143	15.4952	0.7788	2859.0315
30	16.5961	12.5186	0.8509	1258.5807
35	15.8864	11.5138	0.8634	1609.8188
40	**14.1937**	**10.8627**	**0.8909**	**930.9088**
45	15.3471	11.2611	0.8725	1408.0430
50	16.0672	12.1437	0.8603	1513.9424
**(b) Weight-decay sensitivity**				
**Weight decay**	**RMSE ↓**	**MAE ↓**	$R^{2}$↑	**NASA Score ↓**
0	15.7581	11.7530	0.8656	1398.5339
$4\times 10^{-4}$	15.6095	11.7148	0.8681	1156.4684
$8\times 10^{-4}$	**14.1937**	**10.8627**	**0.8909**	**930.9088**
$1.6\times 10^{-3}$	15.1040	11.4071	0.8765	1078.8708
**(c) Asymmetric-loss weighting-ratio sensitivity**				
$\lambda_{\mathrm{over}}:\lambda_{\mathrm{under}}$	**RMSE**↓	**MAE**↓	$R^{2}$↑	**NASA Score**↓
1:1	15.5493	11.5063	0.8691	1301.5071
2:1	**14.1937**	**10.8627**	**0.8909**	**930.9088**
3:1	15.1515	11.2241	0.8757	994.0190

**Table 10:** Ablation results of MBQG-Net on FD001 and FD004.

Dataset	Model Variant	RMSE ↓	MAE ↓	$R^{2}$↑	NASA Score ↓
**FD001**	w/o MS-BiTCN	15.3566	10.9600	0.8531	394.9119
	w/o QV Attention	12.7667	9.5971	0.8985	220.7225
	Single-layer BiGRU	13.4229	9.7978	0.8878	283.7718
	Standard MSE	13.6849	10.4543	0.8834	251.3454
	**Full MBQG-Net**	**12.5659**	**9.3952**	**0.9017**	**203.5562**
**FD004**	w/o MS-BiTCN	19.0734	15.0672	0.8031	1153.3289
	w/o QV Attention	21.4017	17.0708	0.7521	1483.8335
	Single-layer BiGRU	18.0691	13.9680	0.8233	1572.8547
	Standard MSE	15.4390	11.3595	0.8710	1666.9019
	**Full MBQG-Net**	**14.1937**	**10.8627**	**0.8909**	**930.9088**

Note: Lower RMSE, MAE, and NASA Score values indicate better performance, whereas higher $R^{2}$ values are better. Bold indicates the best result within each sub-dataset.

**Table 11:** Comparison between QV and conventional QKV attention on FD004 under an approximately matched parameter budget.

Attention	Trainable Params	Attn. Params	Model State (MB)	RMSE ↓	MAE ↓	$R^{2}$↑	NASA ↓	Train Time(s) ↓
QV	**1,169,682**	**197,393**	**4.473**	**14.1937**	**10.8627**	**0.8909**	**930.9088**	**252.01**
QKV	1,169,729	197,440	4.473	15.0615	11.4965	0.8772	1091.4073	261.75

Note: The QKV internal attention dimension was set to 192 to approximately match the parameter budget of QV. Model-state size was calculated from model parameters and registered buffers. Training time denotes the recorded wall-clock time under the same hardware and software environment.

## Data Availability

The data presented in this study are openly available in [NASA Prognostics Data Repository] [https://data.nasa.gov/dataset/cmapss-jet-engine-simulated-data] (accessed on 28 September 2026).
